# An integrated cuckoo search optimizer for single and multi-objective optimization problems

**DOI:** 10.7717/peerj-cs.370

**Published:** 2021-03-11

**Authors:** Xiangbo Qi, Zhonghu Yuan, Yan Song

**Affiliations:** 1 School of Mechanical Engineering, Shenyang University, Shenyang, China; 2 School of Physics, Liaoning University, Shenyang, China

**Keywords:** Cuckoo search algorithm, Integrated cuckoo search algorithm, Differential evolution, Meta-heuristic

## Abstract

Integrating heterogeneous biological-inspired strategies and mechanisms into one algorithm can avoid the shortcomings of single algorithm. This article proposes an integrated cuckoo search optimizer (ICSO) for single objective optimization problems, which incorporates the multiple strategies into the cuckoo search (CS) algorithm. The paper also considers the proposal of multi-objective versions of ICSO called MOICSO. The two algorithms presented in this paper are benchmarked by a set of benchmark functions. The comprehensive analysis of the experimental results based on the considered test problems and comparisons with other recent methods illustrate the effectiveness of the proposed integrated mechanism of different search strategies and demonstrate the performance superiority of the proposed algorithm.

## Introduction

Meta-heuristic algorithm provides a wide space for solving optimization problems from a new perspective. The meta-heuristic algorithms are created by simulating natural processes or phenomena which show infinite charm with its magic. In order to solve all kinds of optimization problems, many novel meta-heuristics algorithms are developed, such as Ant Colony Optimization (ACO) (Dorigo & Gambardella, 1997), Simulated Annealing (SA) (Kirkpatrick, Gelatt & Vecchi, 1983), Genetic Algorithms (GA) (Holland, 1975), Taboo Search (TS) (Al-Sultan & Fedjki, 1997), Particle Swarm Optimization (PSO) (Kennedy & Eberhart, 1995), Artificial Bee Colony (ABC) (Karaboga & Basturk, 2007), Firefly Algorithm (FA) (Yang, 2010), Cuckoo Search (CS) (Yang, 2014), Pathfinder Algorithm (PFA) (Yapici & Cetinkaya, 2019), Dragonfly Algorithm (DA) (Mirjalili, 2016) and Water Cycle Algorithm (WCA) (Sadollah et al., 2015). It can be seen from these documents that meta-heuristic algorithm has spanned over half a century. The meta-heuristic algorithms can be taken into account in three classes: evolutionary based, physical-based and swarm intelligence based. GA is an evolutionary based algorithm which simulates the natural selection and genetic mechanism of Darwinian biological evolution process (Holland, 1975). GA has two major advantages: the ability of dealing with complex problems and parallelism. GA can deal with various types of optimization, but GA converges to local optimum easily. SA is a physical-based algorithm which comes from the principle of solid state annealing (Kirkpatrick, Gelatt & Vecchi, 1983). SA has the advantages of high efficiency and less constrained by initial conditions, but the search time of SA is longer. TS (Al-Sultan & Fedjki, 1997) uses the taboo technique to jump out of local optimum, but TS has strong dependence on the initial solution. A group of seemingly simple ants can find a shorter path to forage, and ants can find food synchronously without centralized control. ACO is a swarm intelligence based algorithm which mimics the foraging behavior of ants (Dorigo & Gambardella, 1997). ACO is suitable for discrete optimization problems, but the search process takes a long time. PSO is a popular swarm intelligence based algorithm which simulates the social behavior of bird flocking or fish schooling (Kennedy & Eberhart, 1995). ABC is a popular swarm intelligence based algorithm by modeling waggle dance and foraging behaviors of real bee colonies (Karaboga & Basturk, 2007). The ABC algorithm divides the bees into three kinds: employed bees, onlooker bees and scout bees. The three kinds of bees cooperate to realize the exploration and exploitation of the solutions. However, ABC is easy to implement and has few control parameters. However, the optimization performance of ABC becomes worse when the problem dimension becomes larger. FA is a newly proposed swarm intelligence algorithm which is based on the idealized behavior of the flashing characteristics of fireflies (Yang, 2010). DA is a swarm intelligence based algorithm which simulates the static and dynamic swarming behaviors of dragonflies and five parameters are needed to control different behaviors (Mirjalili, 2016). DA is inspired by the static and dynamic swarming behaviors of dragonflies. The multi-objective DA (MODA) tends to find very accurate approximations of Pareto optimal solutions with high uniform distribution for multi-objective problems (Mirjalili, 2016). PFA is a relatively new meta-heuristic algorithm inspired by collective movement of animal group and mimics the leadership hierarchy of swarms to find the best food area or prey (Yapici & Cetinkaya, 2019). WCA simulates the circulation of water in nature, especially the process that small streams on the surface gather into large streams and finally flow to the sea. Experimental results on a set of instances demonstrate the validity of multi-objective WCA (MOWCA) for solving multi-objective optimization problems (Sadollah et al., 2015). Recent work has shown that the optimization ability of the CS algorithm is better than that of PSO and GA (Yang & Deb, 2010). In particular, the meta-heuristic optimization algorithm based on population is now making a significant contribution to the optimization of modern engineering problems (Yapici & Cetinkaya, 2019).

The swarm intelligence method proposed in recent years has a certain ability to solve complex problems, but it is still difficult to meet the requirements (robustness, accuracy and stability) in solving complex and large-scale optimization problems. Each algorithm has its advantages and disadvantages. Beyond that, “No Free Lunch” theorems (Wolpert & Macready, 1997) suggest that there is no meta-heuristic algorithm which appropriate for all optimization problems. Many strategies including improving existing algorithms or integrating all kinds of search strategies into one algorithm can get better optimization effects. Good integration requires clever use at the right time and place, which is still an unsolved problem (Ting et al., 2015). In order to improve the optimization performance of CS, an integrated CS optimizer (ICSO) is proposed in this article.

Our contributions can be summarized to four aspects: (1) A two subpopulation structure based on dynamic size is used. (2) The two subpopulations adopt different position updating methods. (3) The scaling factor of step size is reduced by linear decreasing in Lévy flight phase. (4) After the two subpopulations are merged into one population, an individual information exchange mechanism based on DE operation is applied to the whole population.

The structure of the reminder of this article is as follows. The canonical CS and its variants are introduced in the section “CS algorithm and its variants”. The section “The proposed ICSO” proposes several search strategies and presents the proposed ICSO in detail. The section “Experiment and results of ICSO” gives the details of the experiment for single objective problems, presents the experiment results and discusses the results. The section “Experiment and results of MOICSO” presents the details of the experiment for multi-objective problems and the experiment results. Finally, the section “Conclusion” summarizes the proposed algorithm and draws the conclusions.

## CS algorithm and its variants

Cuckoos can’t make nests or brood. When other birds (host birds) go out to look for food, cuckoos lay their eggs in the nest of the host birds and let the host birds raise their children. Of course, before laying eggs, cuckoos remove the host birds’ eggs in order not to be detected by the host birds. Once the chicks are hatched by their foster mothers, they also have the nature to push their nestlings of the host bird itself out of their nests. The canonical CS algorithm mimics the reproduction of the cuckoo birds (Yang, 2014). The CS algorithm supposes that each cuckoo lays only one egg at a time. When the host bird finds an alien egg, it abandons the nest and finds another place to build a new one. The process of cuckoos’ looking for the nest and laying eggs is the process of finding the solution in the D-dimensional space, and the quality of the bird’s nest symbolizes the quality of the solution. Whether an egg (or a nest) can be successfully hatched by the host bird and thrive is the only criterion to judge whether the solution is good or not.

**Figure 1:**
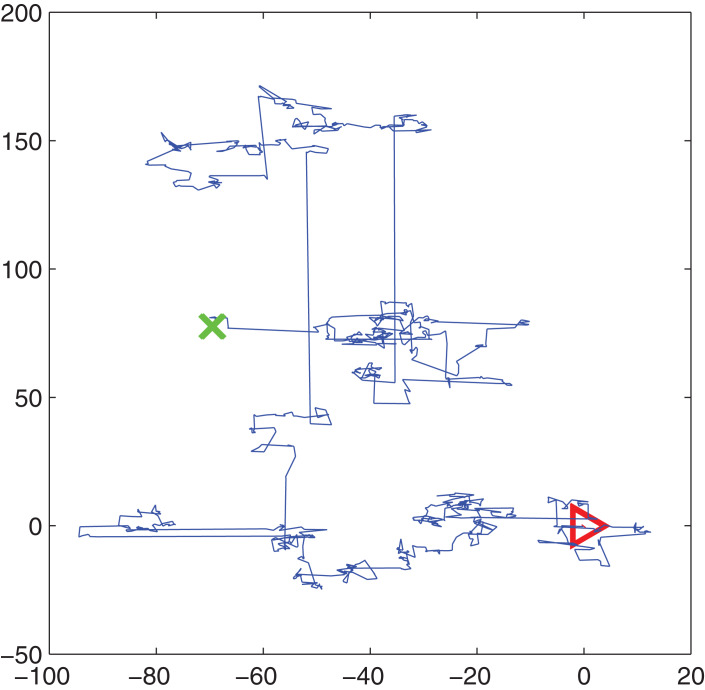
Lévy flight simulation.

In the CS algorithm, there are two ways to update the solutions. One is the cuckoo's way to find the nest to lay eggs, the other is the location path of the host bird to rebuild the nest after finding the foreign bird with a certain probability }{}{P_a} \in \left[ {0,1} \right]. The way of rebuilding the nest’s position can be in Lévy flight or random mode. The cuckoo’s way of finding a nest and laying eggs is to use the Lévy flight according to Eq. (1). Lévy flight provides a random walk whose random step length is drawn from a Lévy distribution according to Eq. (2). An instance of simulation of Lévy flight in 2D plane is shown in Fig. 1. The triangle is the starting point and the cross is the end after several Lévy flights. From Fig. 1, we can see that Lévy flight is a kind of walk between long steps and short steps, which is actually the main feature of the Lévy flight. It is confirmed that many birds in nature follow Lévy’s flight (Viswanathan, 2010).

(1) }{}x_p^{k + 1} = x_p^k + stepSize\; \otimes \; L\acute{e}vy (\rm{\gamma})

(2) }{}L\acute{e}vy ({\rm{\gamma}})\sim u  {t^{ - 1 - {\rm{\gamma}} }},\;0 < {\rm{\gamma}} \le 2

where }{}{\rm \; }{x_p} is the position vector of the *p*th solution, }{}k is the current iteration and }{}stepSize is the step size which should be related to the scales of the problem. \otimes represents entry-wise multiplications.

The pseudo code of CS is listed in Table 1. The termination condition of the CS algorithm may be the maximum cycles or the maximum function evaluation.

**Table 1:** Pseudo code of CS.

1. Initializing the parameters
2. Initializing the population
3. While not meet the stopping criteria
Generate new solutions by Lévy flights
Choose a best nest among new and old solutions
Abandon a fraction of worse solutions using }{}Pa and build new solutions by random walk
Choose a best nest among new and old solutions
Record the best solution
5. End while
6. Post processing

CS uses the whole update and evaluation strategy on solutions. This strategy may deteriorate the convergence speed and the quality of solution due to interference phenomena among dimensions when solving multi-dimension function optimization problems. To overcome this shortage, a dimension by dimension improvement based CS algorithm (DDICS) is proposed (Wang, Yin & Zhong, 2013). The way of rebuilding the nest's position adopts a random mode according to Eq. (3).

(3) }{}x_p^{k + 1} = x_p^k + r\; \otimes {\rm Heaviside}\; \left( {Pa - \varepsilon } \right)\otimes \left( {x_i^k - x_j^k} \right)

where }{}{x_p} is the position vector of the *pth* solution, }{}k is the current iteration. }{}{P_a} \in \left[ {0,1} \right]. \;r\ {\rm and}\ {\rm{\varepsilon}} are random numbers uniformly generated in the range of [0,1]. \otimes represents entry-wise multiplications. }{}{x_i} is the position vector of the *i*th solution, }{}{x_j} is the position vector of the *j*th solution.

The article (Zhang, Ding & Jia, 2019) proposed a hybrid CSDE algorithm. In the CSDE process, population updates are done by partitioning and combining. In the division process, the entire population is divided into two equal subgroups randomly. The two subgroups have the same number of individuals. After all individuals complete the search process, the two subgroups obtained by CS and DE are combined into one group so that individuals can share location information across the search space. The goal of this operation is to enable each individual to find the best solution in less time and maintain its useful information.

The parameter }{}{P_a} plays an important role which maintains balance between the local random walk and the global random walk. In the article (Mareli & Twala, 2018), three CS algorithms including CSLI, CSEI and CSPI are proposed on dynamic changing }{}{P_a} value as the number of CS iterations increases. CSLI uses linear increasing }{}{P_a} value. CSEI uses exponential increasing }{}{P_a} value. CSPI uses power increasing }{}{P_a} value. Simulation results show that CSEI is more effective in finding global minimum values of the sample to test functions used.

## The proposed ICSO

To achieve good optimization performance with higher convergence speed and avoid falling into local optimal solutions, an ICSO is proposed by introducing multiple strategies into the original CS algorithm.

### Two subpopulation structure

**Figure 2:**
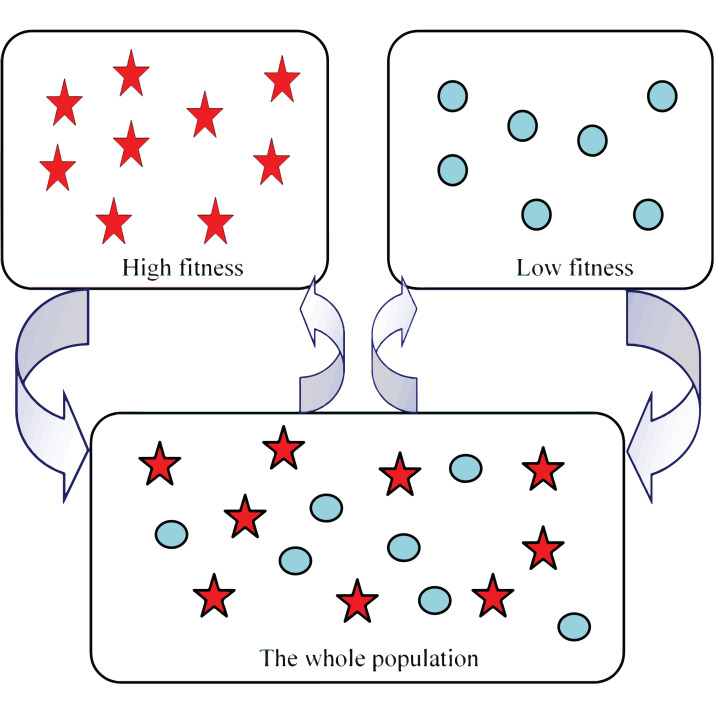
Population structure in ICSO.

Many species boast complex societies with multiple levels of communities. A common case is when two dominant levels exist, one corresponding to leaders and the other consisting of followers (Ferdinandy, Ozogány & Vicsek, 2017). In this paper we change the structure of population of the CS algorithm. At each iteration, the population is divided into two subpopulations according to the individual fitness. Those who own better fitness form high fitness subpopulation while those who own worse fitness form low fitness subpopulation, seen from Fig. 2. The pentagram symbols represents the individuals with high fitness and the circle symbols represents the individuals with low fitness, as shown in Fig. 2.

The high fitness subpopulation is responsible for exploiting better solutions, while the low fitness subpopulation is responsible for exploring unknown solutions. Exploration plays a major role in the early iterations while exploitation plays a major role in the late iterations. So the size of each subpopulation is dynamically changed as the iteration number increases. The size of the high fitness subpopulation increases with the iteration while the size of the low fitness subpopulation decreases with the iteration. In the whole iteration process, the size of the whole population remains unchanged. The value of }{}{P^L} indicates the proportion of the low fitness subpopulation in the whole population. The value of }{}{P^L} decreases linearly from }{}P_{\max}^L to }{}P_{\min}^L with the increasing of iteration times, as shown in Eq. (4).

(4) }{}{P^L} = P_{\max}^L - \displaystyle{k \over E}\left( {P_{\max}^L - P_{\min}^L} \right)

(5) }{}{P^H} = 1 - {P^L}

where }{}k is the current iteration and }{}E is the maximum iterations. }{}{P^L} is the proportion of the low fitness subpopulation in the whole population. }{}{P^H} is the proportion of the high fitness subpopulation in the whole population.

The two subpopulations employ different search strategies which can guarantee the diversity. Meanwhile, two subpopulations adopt different search strategies to realize a balanced combination of a local random walk and a global explorative random walk. A lévy flight is employed to exploit the most promising area, so the high fitness population adopts a lévy flight mode. The previous literature has also shown that some of the new solutions should be produced by lévy flight around the best solution obtained so far, which will often speed up the local search (Pavlyukevich, 2007). A random walk method is used to explore the unknown area, so the low fitness population adopts a random walk method. In this paper, a random method is adopted according to Eq. (3).

### Adaptive step size strategy

The step scaling factor (}{}stepSize in Eq. (1)) plays an important role in updating the position. This parameter is related to the scales of the problem. When the variable range increases, the step size should be increased accordingly. When the variable range decreases, the step size should be reduced accordingly.

In addition, it is very important to balance the ability of global exploration and local exploitation in designing a population-based algorithm. Exploration plays a major role at the beginning of the algorithm, and the step size should be larger. In the later stage of the algorithm, exploitation plays a major role and a big step size may make it difficult for the algorithm to converge to the optimal point. So the step size should be smaller in the later stage of the algorithm.

Based on the above two points, in order to make step size adapt to different optimization problems, a self-adaptive step size mechanism for high fitness subpopulation is introduced. The value of the step scaling factor }{}stepSize decreases linearly from }{}ste{p_{\max}} to }{}ste{p_{\min}} with the increasing of iteration times, as shown in the Eq. (6).

(6) }{}stepSize = ste{p_{\max}} - \displaystyle{k \over E}\left( {ste{p_{\max}} - {step_{\min}}} \right)

(7) }{}ste{p_{\max}} = 0.01*range

(8) }{}{step_{\min}} = 0.001*range

where }{}k is the current iteration and }{}E is the maximum iterations. }{}range is the variable range. For problems with different variable ranges in different dimensions, }{}range is set to the maximum value.

### Crossover based on DE operation

Because the two subpopulations adopt different search strategies, the solutions have diversity on the whole, seen from Fig. 2. To take advantage of the diversity of these solutions to find the potential optimal solution, the two subpopulations are combined into a population and a crossover operation is applied to the population.

Differential evolution (DE) proposed by Storn & Price (1997) performs very well in convergence (Gong, Cai & Ling, 2011). Especially, DE has a good performance on searching the local optima and good robustness (Mallipeddi et al., 2011). Based on the fast convergence speed of DE, a crossover phase based on DE is added after the two population are combined into a whole population. From the perspective of biodiversity, this phase can increase the individual information exchange. The steps of the crossover operation are given in Table 2.

(9) }{}{x_{ij}} = {x_{rj}} + F\left( {{x_{pj}} - {x_{qj}}} \right)

where }{}i, *r*,\; p,\; q are random numbers between 1 and the size of population and mutually different, \; j is a dimension index between [1, *D*]. *F* is the differential weight in the range of [0, 2].

**Table 2:** Crossover operation.

1. Set the parameter values:\; CR and }{}F
2. For each position
3. For each dimension
4. Produce a random number \rm \theta
5. if (\rm \theta \lt CR) then update the value of the current dimension using Eq. (9)
6. End for
7. Calculate the fitness of the new position
8. Greedy selection between the new position and the old position
9. End for

**Figure 3:**
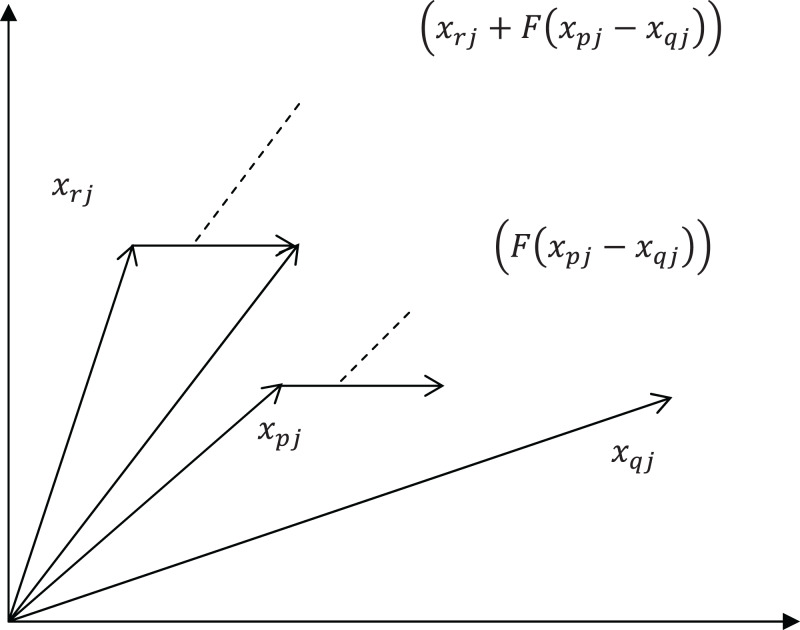
A two-dimensional example for producing new position.

In the crossover operation, the differential mutation operator is the main operation. Figure 3 shows a two-dimensional example for producing new position using Eq. (9). The DE operator is employed to produce a self-adaptive mechanism to change the search range. In the initial stage, individual differences are large, and the algorithm searches in a wide range. At the later stage of algorithm, the population is in a state of convergence, and the individual difference is small. The algorithm searches in a narrow range in the later period of the optimization. Therefore, using DE operator alone will cause the diversity to disappear quickly.

In the proposed ICSO, the initial population is divided into two subpopulations according to the fitness. The two subpopulations update their positions according to different search methods to ensure the diversity of the solutions. Crossover operation is applied after the two subpopulations are merged. After the crossover operation, the whole population is divided into two subpopulations according to their fitness. The above process goes on and on until the end of the algorithm iteration.

### The proposed algorithm for single-objective problems

**Figure 4:**
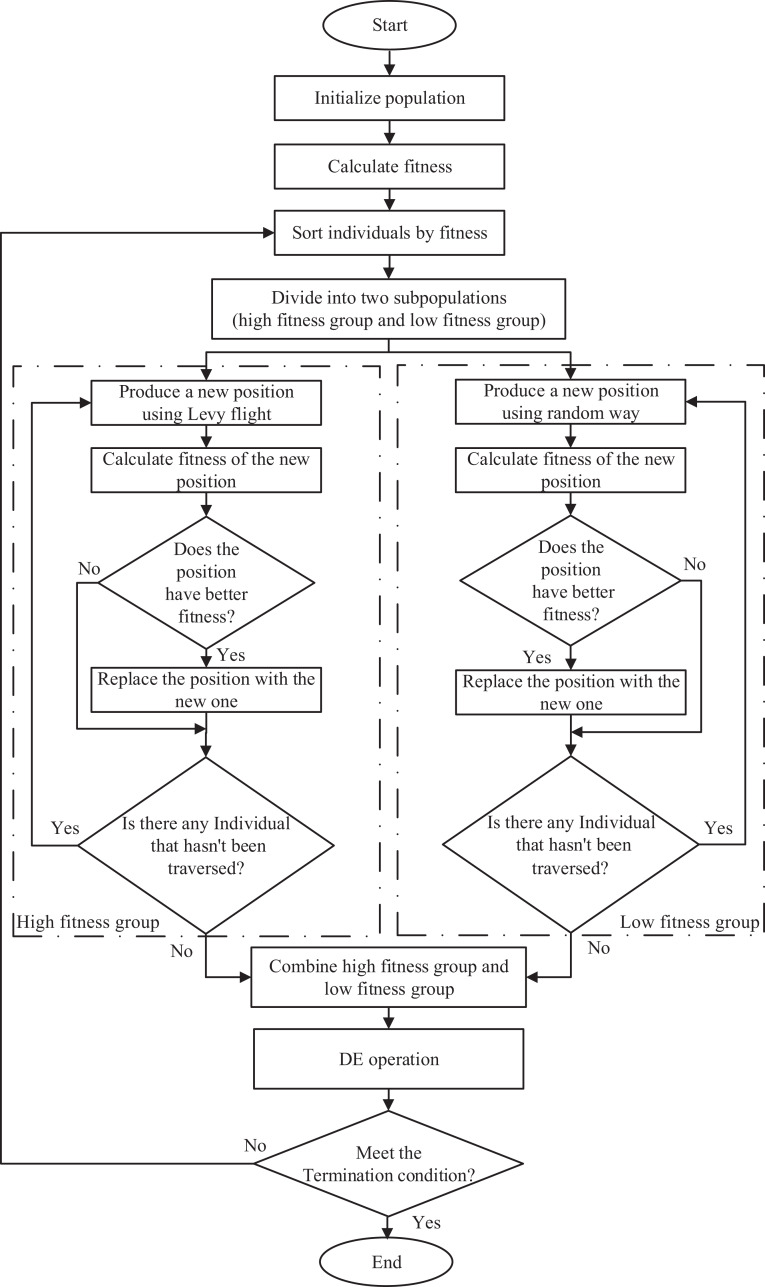
Flowchart of ICSO.

The procedure of ICSO is shown in Table 3 and the flowchart of ICSO is summarized in Fig. 4.

**Table 3:** Pseudo code of the proposed ICSO.

Step 1: Initialization phase:
randomly produces a number of positions according to the Eq. (10)
Step 2: Division phase:
Step 2.1: Rank the positions according to fitness
Step 2.2: Divide population into two subpopulations: high fitness subpopulation and low fitness subpopulation
Step 3: High fitness subpopulation phase:
Step 3.1: Produce step scaling factor according to the Eq. (6)
Step 3.2: Produce new positions according to the Eq. (1)
Step 3.3: Calculate the fitness of new positions
Step 3.4: Greedy selection between new positions and old positions
Step 4: Low fitness subpopulation phase:
Step 4.1: Produce new positions according to the Eq. (3)
Step 4.2: Calculate the fitness of new positions
Step 4.3: Greedy selection between new positions and old positions
Step 5: Combination phase:
Combine high fitness subpopulation and low fitness subpopulation into a whole population
Step 6: Crossover phase:
DE operation
Step 7: If meet termination condition, stop the procedure; otherwise, go to step 2
Step 8: post processing

From Table 3, we can see that initialization phase is responsible for initializing the solution. Eq. (10) gives the way to initialize the solution.

(10) }{}x_{i,j} = x_{j}^{\min} + rand [0,1]\left(x_{j}^{\max}-x_{j}^{\min}\right)

where }{}{x_{i,j}} (}{}1 \le i \le N,1 \le j \le D) represents the initial value of the *j*th variable of *i*th agent. }{}N and *D* are the size of population and the number of variables, respectively. }{}{\rm \; }x_j^{max} and }{}x_j^{min} denote the upper and lower bounds of the *j*th parameter, respectively.

### The proposed algorithm for multi-objective problems

Many problems in real life are multi-objective. Without loss of generality, the mathematical expression of the multi-objective problem is as follows:

(11) }{}{\rm Minimize}\; f\left( x \right) = \left\{ {{f_i}\left( x \right)} \right\},i = 1,\; 2, \ldots ,m\; \;

(12) }{}{\rm Subject\; to}:{\rm \; }{h_j}\left( x \right) = 0,\; j = 1,\; 2, \ldots ,\; p{\rm \; }

(13) }{}{g_k}\left( x \right) \le 0,k = 1,\; 2, \ldots ,\; q

(14) }{}x = \left( {{x_1},\; {x_2}, \ldots ,\; {x_n}} \right)

(15) }{}lo{w_\alpha } \le {x_\alpha } \le hig{h_\alpha },\alpha = 1,\; 2, \ldots ,n

where }{}m > 1, }{}f\left( x \right) is called multi-objective functions. *p* is the number of equality constraints. }{}q is the number of inequality constraints. }{}lo{w_\alpha } is the lower bound of the }{}{\rm \alpha th} variable. }{}high_{\rm\alpha } is the upper bound of the \rm \alpha th variable.

The Pareto front method is used to solve the multi-objective problems through the calculation of Pareto front. Let }{}U = \left( {{u_1},{u_2},\; \ldots ,{u_n}} \right), \; V = \left( {{v_1},{v_2},\; \ldots ,{v_n}} \right) are two vectors. }{}U is said to dominate }{}V if and only if }{}U is partially less than }{}V in objective space. }{}{x^*} is said to be a Pareto optimal solution if and only if any other solutions can’t be detected to dominate }{}{x^*}. Pareto optimal solutions are also called non-dominated solutions. A set of Pareto optimal solution is called Pareto optimal front.

In order to evaluate the performance of multi-objective optimization algorithm quantitatively, two kinds of performance metrics are given here. One is convergence metric, the other is diversity metric convergence (Deb et al., 2002). The generation distance (GD) measure is defined as the criterion of convergence, which is calculated according to Eq. (16).

(16) }{}{\rm GD} = \sqrt {\displaystyle{1 \over {{n_{pf}}}}\mathop \sum \nolimits_{i = 1}^{{n_{pf}}} d_i^2}

where }{}{n_{pf}} is the number of optimal solutions in the obtained Pareto front. }{}{d_i} is the Euclidean distance from each non dominated solution to the nearest true Pareto front solution.

**Figure 5:**
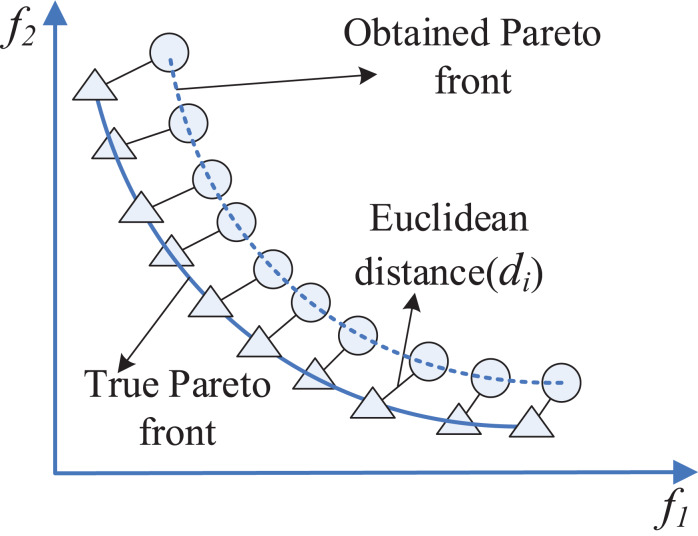
Schematic view of GD criterion.

The computing of GD is illustrated by Fig. 5 where triangles and circles correspond to the solutions on the true Pareto front and the obtained Pareto front, respectively.

The metric of spacing (*S*) measure is defined as the criterion of diversity, which is calculated according to Eq. (17).

(17) }{}S = \sqrt {\displaystyle{1 \over {{n_{pf}} - 1}}\mathop \sum \nolimits_{i = 1}^{{n_{pf}}} {{\left( {{d_i} - \bar d} \right)}^2}}

where }{}{n_{pf}} is the number of optimal solutions in the obtained Pareto front. }{}{d_i} is the crowding distance of the }{}i-th solution. \bar d is the average of all }{}{d_i}.

**Figure 6:**
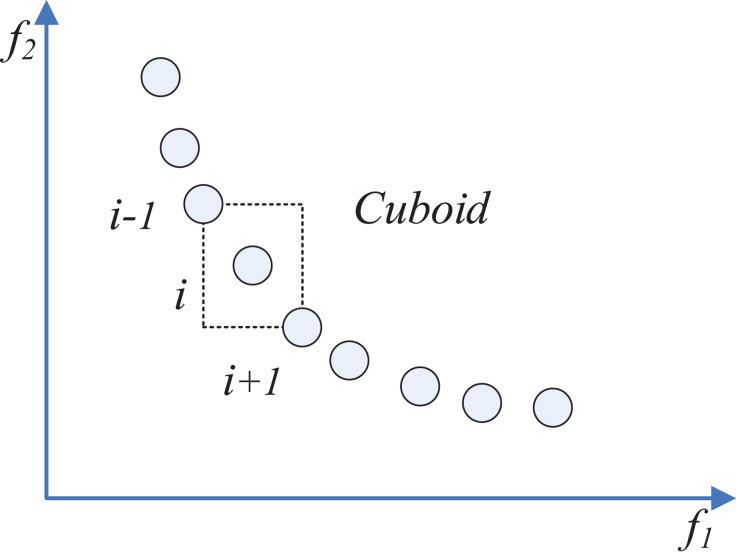
Schematic view of crowding-distance.

The computing of crowding-distance is illustrated by Fig. 6. The crowding distance of the }{}i-th solution is the average side length of the rectangle formed by the dotted line (Deb et al., 2002).

For the advantages of the ICSO, this article proposed a multi-objective ICSO (MOICSO) which can deal with multi-objective optimization problems. The main steps of the MOICSO are shown in Table 4.

**Table 4:** Pseudo code of the proposed MOICSO.

Step 1: Initialization phase:
Randomly produces a number of positions according to the Eq. (10)
Calculate the fitness of each position
Step 2: Non-dominated sorting
Step 3: Crowding distance calculating
Step 4: Division phase:
Step 4.1: Rank the positions according to crowding distance
Step 4.2: Divide population into two subpopulations: high fitness subpopulation and low fitness subpopulation
Step 5: High fitness subpopulation phase:
Step 5.1: Produce step scaling factor according to the Eq. (6)
Step 5.2: Produce new positions according to the Eq. (1)
Step 5.3: Calculate the fitness of new positions
Step 5.4: Greedy selection between new positions and old positions
Step 6: Low fitness subpopulation phase:
Step 4.1: Produce new positions according to the Eq. (3)
Step 4.2: Calculate the fitness of new positions
Step 4.3: Greedy selection between new positions and old positions
Step 7: Combination phase:
Combine high fitness subpopulation and low fitness subpopulation into a whole population
Step 8: Crossover phase:
DE operation
Step 9: Non-dominated sorting
Step 10: Crowding distance calculating
Step 11: If meet termination condition, stop the procedure; otherwise, go to step 4
Step 12: post processing

It is worth noting that compared with ICSO, MOICSO has four more phases. Non-dominated sorting phase is to calculate the non-dominated solutions in the whole population. Crowding distance calculating procedure has been illustrated by Fig. 6.

## Experiment and results of ICSO

In this research, the number of function evaluations is used as the termination condition of each algorithm. All algorithms were coded in Matlab.

### Benchmark problems

Numerical optimization problem solving capability of ICSO is examined by using 24 classic benchmark problems (Civicioglu & Besdokb, 2019; Karaboga & Akay, 2009), *f*_1_–*f*_24_. All benchmark functions used their standard ranges. Detailed mathematical definitions of *f*_1_–*f*_24_ are given in Table 5.

**Table 5:** Benchmark functions.

Name	Function	Limits
Sphere (*f*_1_)	}{}{f_1} = \mathop \sum \limits_{i = 1}^D {x_i^2}	}{}{\left[ { - 5.12,5.12} \right]^D}
Rosenbrock (*f*_2_)	}{}{f_2} = \sum \limits_{i = 1}^D \left(100\left(x_i^2-x_{i+1}\right)+(1-x_i)^2 \right)	}{}{\left[ { - 15,15} \right]^D}
Quadric (*f*_3_)	}{}{f_3} = \sum \limits_{i = 1}^D \left(\sum_{j=1}^{i}x_j\right)^2	}{}{\left[ { - 10,10} \right]^D}
Sinproblem (*f*_4_)	}{}{f_m} = 10{\rm sin}{^2}{\rm \pi } {x_1} + \mathop \sum \limits_{i = 1}^{D - 1} {\left( {{x_i} - 1} \right)^2}\left( {1 + 10{\rm sin}{^2}{\rm \pi } {x_{i + 1}}} \right)	}{}{\left[ { - 10,10} \right]^D}
	}{}{f_4} = \displaystyle{\pi \over D}\left\{ {{f_m} + {{\left( {{x_D} - 1} \right)}^2}} \right\}	
Sumsquares (*f*_5_)	}{}{f_5} = \sum \limits_{i = 1}^D {ix}_i^2	}{}{\left[ { - 10,10} \right]^D}
Powers (*f*_6_)	}{}{f_6} = \mathop \sum \limits_{i = 1}^D {\left| {{x_i}} \right|^{i + 1}}	}{}{\left[ { - 1,1} \right]^D}
Zakharov (*f*_7_)	}{}{f_7} = \sum\limits_{i = 1}^D {x_i^2} + {\left(\sum\limits_{i = 1}^D {0.5i{x_i}} \right)^2} + {\left(\sum\limits_{i = 1}^D {0.5i{x_i}} \right)^4}	}{}{\left[ { - 5,10} \right]^D}
Penalized (*f*_8_)	}{}{f_{t}} = \displaystyle{{\rm \pi } \over {D}}\left\{ {10{{\rm sin}^2}\left( {{\rm \pi }{{x}_1}} \right) + \mathop \sum \limits_{{i} = 1}^{{D} - 1} {{\left( {{{x}_{ i}} - 1} \right)}^2}\left[ {1 + 10{{\rm sin}^2}\left( {{\rm \pi }{{x}_{{i} + 1}}} \right)} \right]} \right\}	}{}{\left[ { - 50,50} \right]^D}
	}{}{f_8} = \displaystyle{{\rm \pi } \over {D}}\left\{ {{{f}_{t}} + {{\left( {{{x}_{D}} - 1} \right)}^2}} \right\} + \mathop \sum \limits_{{i} = 1}^{D} {u}\left( {{{x}_{i}},10,100,4} \right)	
Whitley (*f*_9_)	}{}{f_{t}} = \displaystyle{{{{\left( {100{{\left( {{x}_{i}^2 - {{x}_{j}}} \right)}^2} + {{\left( {1 - {{x}_{j}}} \right)}^2}} \right)}^2}} \over {4,000}}	}{}{\left[ { - 10,10} \right]^D}
	}{}{f_9} = \mathop \sum \limits_{i = 1}^D \mathop \sum \limits_{{j} = 1}^{D} {{f}_{t}} - {\rm cos}\left( {100{{\left( {{x}_{i}^2 - {{x}_{j}}} \right)}^2} + {{\left( {1 - {{x}_{j}}} \right)}^2} + 1} \right)	
Powell (*f*_10_)	}{}{f_t} = {\left( {{x_{4i - 3}} + 10{x_{4i - 2}}} \right)^2} + 5{\left( {{x_{4i - 1}} - {x_{4i}}} \right)^2}	}{}{\left[ { - 4,5} \right]^D}
	}{}{f_{10}} = \mathop \sum \limits_{i = 1}^{D/4} \left[ {{f_t} + {{\left( {{x_{4i - 2}} - 2{x_{4i - 1}}} \right)}^4} + 10{{\left( {{x_{4i - 3}} - {x_{4i}}} \right)}^4}} \right]	
Solomon (*f*_11_)	\Vert x \Vert = \sqrt {\mathop \sum \nolimits_{i = 1}^D x_i^2}	}{}{\left[ { - 100,100} \right]^D}
	}{}{f_{11}} = 1 - \cos\left( {2{\rm \pi} \|x\| + 0.1\|x\|} \right)	
	}{}{w_i} = 1 + \displaystyle{{{x_i} - 1} \over 4}	
Levyfunc (*f*_12_)	}{}{f_t} = \mathop \sum \limits_{i = 1}^{D - 1} {\left( {{w_i} - 1} \right)^2}\left[ {1 + 10{\sin^2}\left( {{\rm \pi} {w_i} + 1} \right)} \right]	}{}{\left[ { - 10,10} \right]^D}
	}{}{f_{12}} = {\sin^2}\left( {{\rm \pi} {w_1}} \right) + {f_t} + {\left( {{w_d} - 1} \right)^2}\left[ {1 + {\sin^2}\left( {2{\rm \pi} {w_d}} \right)} \right]	
Schwefel_1_2 (*f*_13_)	}{}{f_{13}} = \mathop \sum \limits_{i = 1}^D {\left( {\mathop \sum \limits_{i = 1}^i {x_j}} \right)^2}	}{}{\left[ { - 100,100} \right]^D}
Schwefel_2.22 (*f*_14_)	}{}{f_{14}} = \sum\limits_{i = 1}^D {\left| {{x_i}} \right|} + \prod\limits_{i = 1}^D {\left| {{x_i}} \right|}	}{}{\left[ { - 10,10} \right]^{D}}
Schwefel (*f*_15_)	}{}{f_{15}} = D \cdot 418.9829 + \sum\limits_{i = 1}^D { - {x_i}} \sin (\sqrt {\left| {{x_i}} \right|} )	}{}{\left[ { - 500,500} \right]^{D}}
Weierstrass (*f*_16_)	}{}{f_{16}} = \mathop \sum \limits_{{i} = 1}^{D} \left[ {\mathop \sum \limits_{{k} = 0}^{20} {{0.5}^{k}}\cos \left( {2{\rm \pi }{3^{k}}\left( {{x} + 0.5} \right)} \right) - {D}\mathop \sum \limits_{{k} = 0}^{20} {{0.5}^{k}}{\cos}\left( {{\rm \pi }{3^{k}}} \right)} \right]	}{}{\left[ { - 0.5,0.5} \right]^D}
Rastrigin (*f*_17_)	}{}{f_{17}} = \sum\limits_{i = 1}^D {(x_i^2 - 10\cos (2\pi {x_i}) + 10)}	}{}{\left[ { - 15,15} \right]^{\rm D}}
Ackley (*f*_18_)	}{}{f_{m}} = 20 + e - 20\exp \left( { - 0.2\sqrt {\displaystyle{1 \over D}\mathop \sum \nolimits_{i = 1}^D x_i^2} } \right)	}{}{\left[ { - 32.768,32.768} \right]^{ D}}
	}{}{f_{18}} = {{f}_{m}} - \exp\left( {\displaystyle{1 \over D}\mathop \sum \nolimits_{i = 1}^D \cos\left( {2\pi {x_i}} \right)} \right)	
Griewank (*f*_19_)	}{}{f_{19}} = \displaystyle{1 \over {4,000}}\left(\sum\limits_{i = 1}^D {x_i^2} \right) - \left(\prod\limits_{i = 1}^D {\cos \left(\displaystyle{{{x_i}} \over {\sqrt i }}\right)} \right) + 1	}{}{\left[ { - 600,600} \right]^{ D}}
Rot_schwefel (*f*_20_)	}{}{f_{20}} = {f_{15}}(y),y = M \times x	}{}{\left[ { - 500,500} \right]^{ D}}
Rot_weierstrass (*f*_21_)	}{}{f_{21}} = {f_{16}}\left( y \right),y = M \times x	}{}{\left[ { - 0.5,0.5} \right]^D}
Rot_rastrigin (*f*_22_)	}{}{f_{22}} = {f_{17}}(y),y = M \times x	}{}{\left[ { - 15,15} \right]^{D}}
Rot_ackley (*f*_23_)	}{}{f_{23}} = {f_{18}}(y),y = M \times x	}{}{\left[ { - 32.768,32.768} \right]^{D}}
Rot_griewank (*f*_24_)	}{}{f_{24}} = {f_{19}}(y),y = M \times x	}{}{\left[ { - 600,600} \right]^{ D}}

Functions }{}{f_{20}}–}{}f_{24} are four rotated functions (Liang et al., 2006). The fitness of the rotated function is calculated with a rotated variable }{}y instead of }{}x. The rotated variable }{}y is calculated by the original variable }{}x left multiplied an orthogonal matrix. The orthogonal matrix is calculated using Salomon’s method (Salomon, 1996).

### Parameter study

The influences of the parameters including }{}({f_7}) and }{}({f_8}) are experimented in this section. The dimension of the test functions including Sphere, Powers, Ackley and Griewank is set to 30. The population size of each algorithm is set to 50. The computational cost of each experiment was set to 100,000 function evaluations. Each test problem is solved by assigning different values to the two parameters in 30 independent runs. The numerical results in terms of the mean results (Mean), standard deviation (Std), the best result (Best) and the worst result (Worst) of the optimal function value were given in Tables 6–9. The results demonstrate that ICSO performs best on all test functions when both the parameter }{}CR and }{}F are set to 0.1. Therefore, these two parameters are set to 0.1 in the next experiments.

**Table 6:** Results of ICSO on Sphere with different CR and F. In bold are the best results.

CR	F	Mean	Std	Best	Worst
**0.1**	**0.1**	**1.609372E−025**	**2.559493E−025**	**8.182012E−027**	**1.335411E−024**
0.5	0.1	1.079669E−018	5.781429E−018	1.774286E−030	3.168279E−017
0.9	0.1	1.348758E−004	2.669555E−004	1.730580E−010	9.801879E−004
0.1	0.5	2.188021E−018	1.294737E−018	7.106979E−019	5.847799E−018
0.5	0.5	8.248026E−020	6.111145E−020	1.259383E−020	2.580927E−019
0.9	0.5	7.013024E−019	4.406099E−019	1.920019E−019	1.831575E−018
0.1	0.7	2.889654E−014	1.768001E−014	1.126885E−014	8.171438E−014
0.5	0.7	2.093975E−011	8.414275E−012	1.009346E−011	4.587659E−011
0.9	0.7	7.961881E−010	4.865786E−010	1.690856E−010	2.183528E−009

**Table 7:** Results of ICSO on Powers with different CR and F.

CR	F	Mean	Std	Best	Worst
**0.1**	**0.1**	**8.274820E−069**	**4.234084E−068**	**2.341900E−094**	**2.318979E−067**
0.5	0.1	2.533804E−011	5.583066E−011	3.938471E−029	2.634223E−010
0.9	0.1	6.480953E−010	9.583368E−010	5.605142E−015	4.288309E−009
0.1	0.5	5.663635E−062	1.078139E−061	2.652438E−065	5.227871E−061
0.5	0.5	1.184799E−054	3.220997E−054	4.349645E−058	1.728686E−053
0.9	0.5	5.237663E−043	2.823356E−042	5.040580E−054	1.547070E−041
0.1	0.7	4.996276E−048	8.676584E−048	1.881430E−051	2.946174E−047
0.5	0.7	1.151732E−032	4.406950E−032	1.659515E−035	2.407790E−031
0.9	0.7	5.605934E−028	2.765412E−027	2.159583E−031	1.518559E−026

**Note:**

Best results are shown in bold.

**Table 8:** Results of ICSO on Ackley with different CR and F.

CR	F	Mean	Std	Best	Worst
**0.1**	**0.1**	**1.355183E−012**	**8.013313E−013**	**5.728751E−013**	**3.457679E−012**
0.5	0.1	6.488290E−002	2.469753E−001	1.643130E−013	9.931908E−001
0.9	0.1	6.457084E−001	4.954536E−001	1.116077E−006	1.778300E+000
0.1	0.5	1.007639E−008	2.395377E−009	6.108604E−009	1.704621E−008
0.5	0.5	1.742065E−009	6.627207E−010	7.913927E−010	3.717433E−009
0.9	0.5	5.512860E−009	2.304299E−009	1.988494E−009	1.034997E−008
0.1	0.7	1.271131E−006	3.403827E−007	7.282554E−007	1.877949E−006
0.5	0.7	3.932920E−005	1.107632E−005	1.989003E−005	6.887054E−005
0.9	0.7	2.388952E−004	7.178060E−005	1.203356E−004	3.868039E−004

**Note:**

Best results are shown in bold.

**Table 9:** Results of ICSO on Griewank with different CR and F.

CR	F	Mean	Std	Best	Worst
**0.1**	**0.1**	**1.480297E−017**	**4.820283E−017**	**0.000000E+000**	**2.220446E−016**
0.5	0.1	6.777109E−003	2.070448E−002	0.000000E+000	7.093749E−002
0.9	0.1	1.153069E−001	1.037140E−001	5.140760E−010	3.447163E−001
0.1	0.5	2.297496E−013	5.095175E−013	3.552714E−015	2.130962E−012
0.5	0.5	6.202446E−015	2.459358E−014	0.000000E+000	1.233458E−013
0.9	0.5	3.456494E−015	6.899648E−015	0.000000E+000	2.287059E−014
0.1	0.7	3.074112E−009	6.409751E−009	7.266598E−011	2.459320E−008
0.5	0.7	5.636615E−007	1.954695E−006	9.578553E−009	1.052754E−005
0.9	0.7	9.679650E−006	1.646266E−005	4.156234E−007	6.281005E−005

**Note:**

Best results are shown in bold.

### Comparison with other algorithms

In order to compare the performance of ICSO, DDICS (Wang, Yin & Zhong, 2013), DE/rand (Storn & Price, 1997), CSDE (Zhang, Ding & Jia, 2019) and CSEI (Mareli & Twala, 2018) were employed for comparison. DE/rand is a classical population-based algorithm. Here DE/rand stands for DE/rand/1/bin (Storn & Price, 1997). Detailed descriptions of DDICS, CSDE and CSEI are given in “CS Algorithm and its Variants”.

The population size of ICSO and DDICS was 50. Dimensions of }{}{f_1}–}{}{f_9} and }{}{f_{11}}–}{}{f_{24}} are selected as 50. Dimensions of }{}{f_{10}} is selected as 24. The maximum evaluation count for functions }{}{f_1}–}{}{f_{24}} is 200,000. }{}P_{\max}^L\; {\rm and}\; P_{\min}^L are set to 0.9 and 0.2 respectively. To gather significant statistical results, each algorithm carried out 30 independent runs. For the other specific parameters for involved algorithms, we can follow parameters settings of the original literatures of DDICS (Wang, Yin & Zhong, 2013), CSEI (Mareli & Twala, 2018), DE/rand and CSDE (Zhang, Ding & Jia, 2019).

**Figure 7:**
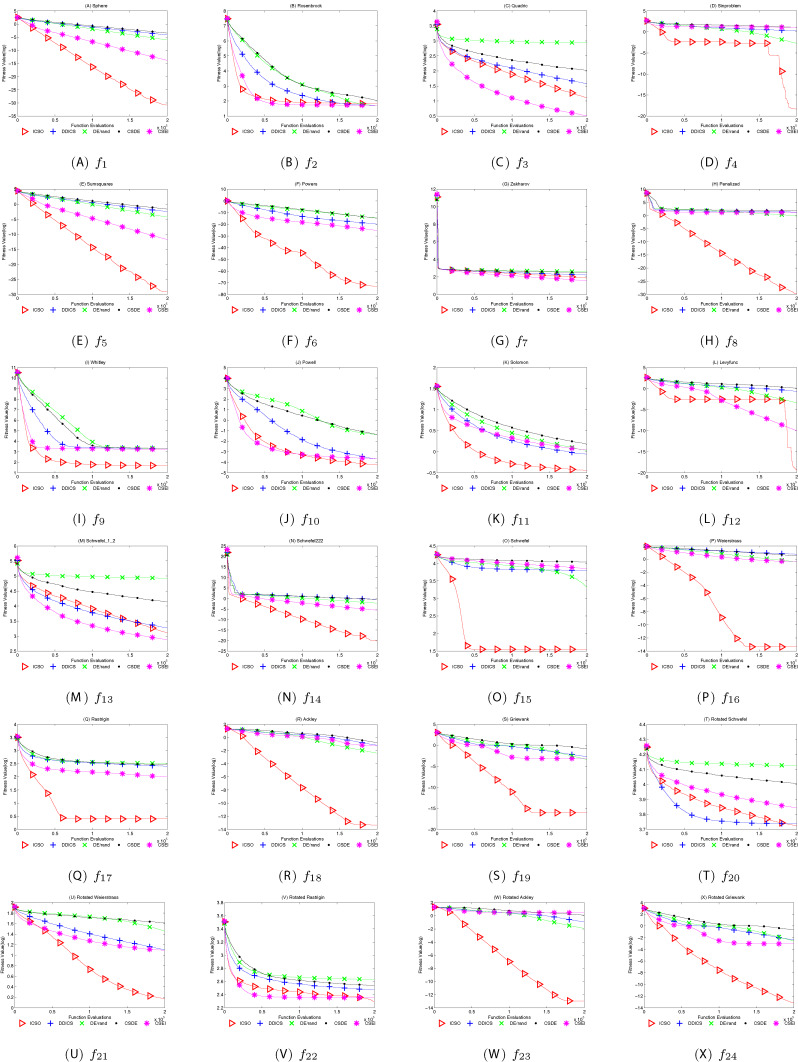
The mean best function value profiles of ICSO, DDICS, CSDE, CSEI and DE/rand. (A–X) Convergence curves of ICSO, DDICS, CSDE, CSEI and DE/rand.

The results of our experiments are illustrated in Table 10 and the average convergence curves obtained for ICSO, DDICS, CSDE, CSEI and DE/rand are depicted in Fig. 7. *M* stands for the mean value and *S* stands for the standard deviation. *B* stands for the best value and *W* stands for the worst value. *R* stands for the performance order of the different algorithms on each standard function. The best mean values obtained on each function are marked as bold. It is obvious that ICSO performed best on most functions.

**Table 10:** Results comparison of different optimal algorithms.

Func.		ICSO	DDICS	CSDE	CSEI	DE/rand
*f*_1_	M	**1.690822E−031**	7.434447E−005	3.632333E−004	1.454569E−014	8.282742E−007
	S	**8.840733E−031**	3.949045E−005	7.745479E−005	1.082064E−014	1.569209E−007
	B	**2.013494E−035**	2.439431E−005	1.753063E−004	2.775412E−015	5.379655E−007
	W	**4.849069E−030**	1.933459E−004	5.281880E−004	5.186465E−014	1.293296E−006
	R	**1**	4	5	2	3
*f*_2_	M	6.072458E+001	**5.032816E+001**	1.102890E+002	5.171790E+001	5.070120E+001
	S	3.405947E+001	**9.117326E+000**	2.439360E+001	2.076860E+001	1.348960E+000
	B	7.224458E+000	**4.602451E+001**	7.785160E+001	3.457020E+001	4.871670E+001
	W	1.521801E+002	**9.642114E+001**	1.788330E+002	9.602550E+001	5.393890E+001
	R	4	**1**	5	3	2
*f*_3_	M	1.279231E+001	3.738756E+001	1.048900E+002	**3.229244E+000**	8.671876E+002
	S	3.177691E+000	6.886322E+000	1.610203E+001	**7.304960E−001**	8.161474E+001
	B	7.277044E+000	2.532631E+001	8.002669E+001	**2.071841E+000**	6.610512E+002
	W	2.413531E+001	5.422825E+001	1.387001E+002	**5.750541E+000**	9.733504E+002
	R	2	3	4	**1**	5
*f*_4_	M	**4.572794E-019**	1.874668E+000	1.218447E+001	1.236526E+001	1.940224E−003
	S	**2.504622E−018**	9.384672E−001	4.917733E+000	5.448729E+000	1.343576E−003
	B	**9.423269E−033**	3.074940E−001	4.730235E+000	2.904683E+000	5.395848E−004
	W	**1.371838E−017**	4.402664E+000	2.520265E+001	2.443012E+001	6.596449E−003
	R	**1**	3	4	5	2
*f*_5_	M	**7.514838E−030**	3.562223E−003	2.376085E−002	1.789593E−012	5.330367E−005
	S	**3.979495E−029**	1.257931E−003	7.057260E−003	1.386500E−012	9.155393E−006
	B	**7.002057E−034**	1.524990E−003	1.008428E−002	4.663644E−013	3.355436E−005
	W	**2.182041E−028**	6.129184E−003	3.733488E−002	6.475331E−012	7.272198E−005
	R	**1**	4	5	2	3
*f*_6_	M	**7.766621E−074**	1.699435E−020	1.654069E−015	6.362256E−026	2.365562E−015
	S	**4.253953E−073**	4.969788E−020	1.585987E−015	3.214170E−025	2.713795E−015
	B	**2.212551E−117**	1.187302E−026	1.390578E−016	3.205505E−035	9.471699E−017
	W	**2.329986E−072**	2.534362E−019	6.408675E−015	1.762635E−024	1.248198E−014
	R	**1**	3	4	2	5
*f*_7_	M	7.500789E+001	2.533525E+002	3.273866E+002	**3.647427E+001**	3.929206E+002
	S	1.524685E+001	1.585340E+001	4.318425E+001	**7.580823E+000**	3.950995E+001
	B	4.432003E+001	2.295845E+002	2.415960E+002	**2.035347E+001**	3.034929E+002
	W	1.039199E+002	2.813324E+002	4.038223E+002	**4.788216E+001**	4.722730E+002
	R	2	3	4	**1**	5
*f*_8_	M	**1.894826E−030**	2.202293E+001	8.878344E+001	1.109721E+001	6.359735E−001
	S	**7.339505E−030**	1.061090E+001	1.118776E+001	7.141229E+000	3.277267E−001
	B	**9.423269E−033**	6.865627E+000	6.799393E+001	2.460831E+000	1.770775E−001
	W	**3.901290E−029**	4.655743E+001	1.152675E+002	2.858832E+001	1.381641E+000
	R	**1**	4	5	3	2
*f*_9_	M	**4.829720E+001**	2.093274E+003	2.129340E+003	1.646785E+003	2.106286E+003
	S	**1.037431E+002**	7.753954E+001	3.958113E+001	9.600529E+001	4.428581E+001
	B	**6.342226E−007**	1.885715E+003	2.033689E+003	1.382389E+003	2.000747E+003
	W	**4.500182E+002**	2.232349E+003	2.200207E+003	1.804301E+003	2.187439E+003
	R	**1**	3	5	2	4
*f*_10_	M	**5.692079E−005**	2.177530E−004	4.236186E−002	2.185153E−004	4.139503E−002
	S	**7.567419E−005**	6.270462E−005	1.292405E−002	1.230560E−004	1.096975E−002
	B	**1.236782E−005**	1.019904E−004	1.798891E−002	6.953727E−005	1.707548E−002
	W	**3.713010E−004**	3.343223E−004	7.913157E−002	5.323149E−004	6.365177E−002
	R	**1**	2	5	3	4
*f*_11_	M	**3.632092E−001**	8.763506E−001	1.504896E+000	1.106541E+000	1.067672E+000
	S	**5.560243E−002**	6.601137E−002	1.312017E−001	1.529777E−001	5.175515E−002
	B	**2.998733E−001**	7.998734E−001	1.204120E+000	8.998734E−001	1.000359E+000
	W	**4.998733E−001**	1.002940E+000	1.802447E+000	1.599873E+000	1.167540E+000
	R	**1**	2	5	4	3
*f*_12_	M	**4.159863E−020**	2.243317E−001	1.250937E+000	9.666004E−011	4.307533E−004
	S	**2.278451E−019**	2.142007E−001	5.747726E−001	1.432857E−010	1.322854E−004
	B	**1.499760E−032**	3.106044E−002	2.678047E−001	4.477200E−013	2.269744E−004
	W	**1.247959E−018**	9.026046E−001	2.696229E+000	5.940079E−010	7.005719E−004
	R	**1**	4	5	2	3
*f*_13_	M	1.285410E+003	1.847284E+003	1.377424E+004	**7.534658E+002**	8.308556E+004
	S	3.692569E+002	3.879304E+002	2.867983E+003	**2.255480E+002**	7.998931E+003
	B	4.048086E+002	1.151343E+003	8.259107E+003	**4.602923E+002**	6.487209E+004
	W	2.043478E+003	2.633676E+003	2.195737E+004	**1.447019E+003**	1.034199E+005
	R	2	3	4	**1**	5
*f*_14_	M	**6.175248E−021**	4.435664E−001	3.846050E−001	2.756024E−006	6.016758E−003
	S	**2.035080E−020**	8.942698E−002	5.330443E−002	1.233858E−006	7.916101E−004
	B	**1.911539E−022**	3.079974E−001	2.730676E−001	1.041940E−006	4.797230E−003
	W	**1.046313E−019**	6.572179E−001	4.850878E−001	6.708609E−006	8.548652E−003
	R	**1**	5	4	2	3
*f*_15_	M	**3.553214E+001**	6.289531E+003	1.098884E+004	7.135581E+003	2.092765E+003
	S	**5.520311E+001**	3.392640E+002	4.390494E+002	6.741034E+002	1.239535E+003
	B	**6.363783E−004**	5.479992E+003	9.581847E+003	4.943241E+003	6.571187E+002
	W	**1.184390E+002**	6.964557E+003	1.142057E+004	8.498809E+003	4.808003E+003
	R	**1**	3	5	4	2
*f*_16_	M	**4.357995E−014**	5.439281E+000	3.221646E+000	3.998280E−001	3.772394E−001
	S	**1.942385E−013**	9.072078E−001	1.923992E−001	2.571573E−001	3.547392E−002
	B	**0.000000E+000**	3.964348E+000	2.863221E+000	3.562026E−002	2.869074E−001
	W	**1.065814E−012**	7.967955E+000	3.604937E+000	1.019892E+000	4.288895E−001
	R	**1**	5	4	3	2
*f*_17_	M	**2.520563E+000**	2.533525E+002	2.911180E+002	9.999019E+001	3.216980E+002
	S	**1.539907E+000**	1.585340E+001	1.011565E+001	1.278406E+001	9.520151E+000
	B	**0.000000E+000**	2.295845E+002	2.683769E+002	7.912906E+001	3.020563E+002
	W	**5.969754E+000**	2.813324E+002	3.178953E+002	1.187638E+002	3.370451E+002
	R	**1**	**3**	4	2	5
*f*_18_	M	**4.766558E−014**	6.622171E−002	1.568679E−001	5.052436E−002	4.060724E−003
	S	**6.581426E−014**	2.764490E−002	2.963107E−002	2.686782E−001	4.587345E−004
	B	**1.509903E−014**	3.765528E−002	9.993845E−002	1.740668E−006	3.203914E−003
	W	**3.774758E−013**	1.795148E−001	2.183519E−001	1.473019E+000	4.954966E−003
	R	**1**	4	5	3	2
*f*_19_	M	**9.621933E−017**	1.972718E−003	1.354911E−001	7.391292E−004	4.343150E−004
	S	**1.814549E−016**	1.102676E−003	3.391925E−002	2.831251E−003	9.771712E−005
	B	**0.000000E+000**	4.216275E−004	8.313643E−002	1.624367E−012	2.284315E−004
	W	**6.661338E−016**	5.107104E−003	2.184895E−001	1.231607E−002	6.441814E−004
	R	**1**	4	5	3	2
*f*_20_	M	**5.288122E+003**	5.468764E+003	1.012564E+004	6.984482E+003	1.332064E+004
	S	**4.930206E+002**	4.193924E+002	4.612909E+002	7.598783E+002	2.980906E+002
	B	**4.052372E+003**	4.835699E+003	9.272974E+003	5.109969E+003	1.256695E+004
	W	**5.885806E+003**	6.372392E+003	1.110335E+004	8.026067E+003	1.383184E+004
	R	**1**	2	4	3	5
*f*_21_	M	**1.479607E+000**	1.269138E+001	4.099588E+001	1.244022E+001	2.885822E+001
	S	**1.588095E+000**	2.205968E+000	3.460469E+000	7.677092E+000	8.004548E+000
	B	**2.229151E−001**	8.999621E+000	3.327664E+001	4.442858E+000	1.930899E+001
	W	**8.835250E+000**	1.812362E+001	4.766046E+001	3.163309E+001	5.103687E+001
	R	**1**	3	5	2	4
*f*_22_	M	**1.967897E+002**	2.963754E+002	3.478769E+002	2.260444E+002	4.261554E+002
	S	**1.552348E+001**	2.342126E+001	2.401303E+001	3.938019E+001	1.694762E+001
	B	**1.538850E+002**	2.156264E+002	2.796065E+002	1.332539E+002	3.729620E+002
	W	**2.291231E+002**	3.454933E+002	3.807513E+002	3.172834E+002	4.504501E+002
	R	**1**	3	4	2	5
*f*_23_	M	**1.055748E−013**	1.137854E−001	1.208615E+000	2.838387E+000	1.179800E−002
	S	**2.972044E−013**	5.688875E−002	2.106246E−001	9.680830E−001	1.296514E−003
	B	**1.509903E−014**	4.076001E−002	6.598466E−001	1.173359E+000	9.293203E−003
	W	**1.663558E−012**	3.092073E−001	1.551999E+000	5.929673E+000	1.437805E−002
	R	**1**	3	4	5	2
*f*_24_	M	**6.693535E−014**	4.117954E−003	2.184338E−001	9.125083E−004	2.729167E−003
	S	**2.300469E−013**	2.757500E−003	5.466496E−002	2.868126E−003	6.417853E−004
	B	**0.000000E+000**	1.385349E−003	1.408811E−001	6.951317E−011	1.756282E−003
	W	**1.218470E−012**	1.209074E−002	3.421869E−001	1.231608E−002	4.059698E−003
	R	**1**	4	5	2	3

**Note:**

The best results are shown in bold.

Table 10 demonstrates that ICSO achieves approving results on most of the twenty-four functions. ICSO has achieved the best mean values except }{}{f_2},\; {f_3},\; {f_7}{\rm \; and\; }{f_{13}}. Compared with the other algorithms, ICSO performs very well when solving the functions }{}{f_1}, \; {f_4}, \; {f_5}, \; {f_6}, \; {f_8}, \; {f_{12}}, \; {f_{14}}, \; {f_{16}}, \; {f_{18}}, \; {f_{19}}, \; {f_{23}} and }{}{f_{24}}.

Particularly, ICSO has a strong ability to mine the optimal value in the later stage of iteration on }{}{f_4} and }{}{f_{12}}, seen from Fig. 7. The performance of ICSO on }{}{f_4} is much similar with it on }{}{f_{12}}. ICSO converged very fast at the beginning and then trapped in local minimum. However, after a certain number of iterations, ICSO jumps out of the local optimum. With multiple search strategies, ICSO keeps sufficient diversity to escape local optimum on functions like }{}{f_4} and }{}{f_{12}}, even at the later stage of algorithm iteration.

As per the results and discussions of this study, we can conclude that ICSO can enhance both exploration and exploitation capabilities effectively. As can be clearly seen from Fig. 7, ICSO seemed to be able to sustain improvement especially on }{}{f_1}, }{}{f_5}, }{}{f_6}, }{}{f_8}, }{}{f_{14}}, }{}{f_{20}}, }{}{f_{21}} and }{}{f_{24}}.

### Statistical analysis

In this section, two statistical evaluation approaches including Iman-Daveport and Holm tests are considered for validating the performance of the proposed ICSO. A detailed introduction of the two statistical evaluation methods can be found in reference (García et al., 2010).

The results of the Iman–Davenport test and Holm tests are given in Tables 11 and 12 respectively. The critical value listed in the Table 11 and the \alpha /i values listed in the Table 12 are with level of 0.05.

**Table 11:** Results of the Iman-Davenport test.

Dimension	Iman-Davenport	Critical value α = 0.05	Significant differences?
50	31.97	2.45–2.52	Yes

**Table 12:** Results of Holm’s test (*f*_1_–*f*_24_).

Algorithm	*z*	*p* value	α/*i*	Significant differences?
CSDE	7.21	5.53E−13	0.0125	Yes
DE/rand	4.65	3.23E−6	0.0167	Yes
DDICS	4.38	1.17E−6	0.025	Yes
CSEI	2.92	0.0035	0.05	Yes

From Table 11, there are significant differences in the ranking of algorithms because the Iman-Davenport values are greater than the critical value. From Table 12, there are significant differences between comparison algorithms and control algorithm (ICSO) because the }{}p values of CSDE, DE/rand, DDICS and CSEI are less than their \alpha /i values.

**Figure 8:**
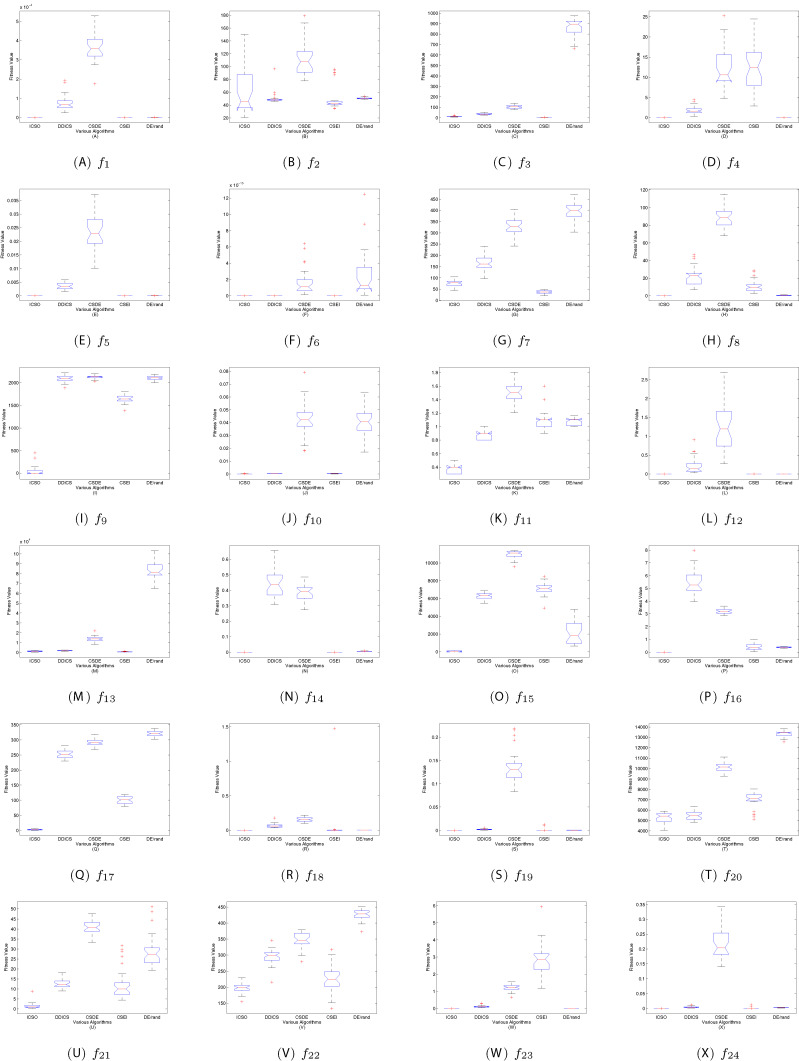
ANOVA results of all algorithms. (A–X) Boxplots of ICSO, DDICS, CSDE, CSEI and DE/rand.

In this article, the validity and stability of ICSO are also studied by analysis of variance (ANOVA). Figure 8 shows the box plots of the statistical performance of all algorithms. The general characteristics of the distribution can be obtained by looking at these box plots. It can be seen clearly from Fig. 8 that ICSO obtains a good compromise solution variance distribution on most test functions.

### Dynamic size of subpopulation

**Figure 9:**
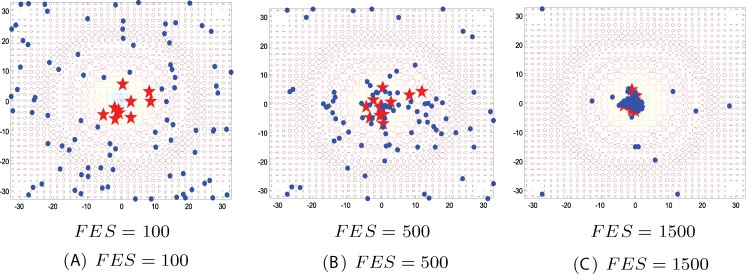
Subpopulation evolution of ICSO model on two-dimensional Ackley function. (A) FES = 100; (B) FES = 500; (C) FES = 1,500.

This simulation is conducted to investigate how the size of subpopulation in the ICSO model changes along with the generations on the benchmark environments. In order to clearly see the change of subpopulation size, the initial population size is set to 100. The subpopulation evolution based on ICSO model was simulated on two-dimensional Ackley function. Figure 9 shows the positions of the individuals and the subpopulation variation in different phases, where each red star represents a position with high fitness and each blue circle represents a position with low fitness.

As can be seen from Fig. 9, the initial positions are distributed randomly over the map defined by the two-dimensional Ackley function at the first stage (FEs = 100). We can directly see from Fig. 9 that with the iteration, the size of high fitness subpopulation increases and the size of low fitness subpopulation decreases. From the first phase (FEs = 100) to third phase (FEs = 1,500), the whole population moves toward the optimal position. The two subpopulations adopt different ways to update the position, and exploit the optimal region and explore the unknown region respectively. Under the collaborative effect of the two subpopulations, ICSO can make the whole population move toward the optimal position.

## Experiment and results of moicso

### Benchmark problems

The test problems includes seven problems which are denoted by ZDT1, ZDT3, ZDT6, DTLZ1, DTLZ2, DTLZ3 and DTLZ6. ZDT1, ZDT3 and ZDT6 have two objective functions. DTLZ1, DTLZ2, DTLZ3 and DTLZ6 have three objective functions.

The ZDT1 problem (Deb et al., 2002) is described as follows:

(18) }{}{\rm ZDT1}:\left\{ {\matrix{ {{\hskip-65pt}{\rm Minimize}\;{f_1}\left( x \right) = {x_1}} \cr {{\rm Minimize}\;{f_2}\left( x \right) = g\left( x \right)\left[ {1 - \sqrt {\displaystyle{{{x_1}} \over {g\left( x \right)}}} } \right]}\cr {g\left( x \right) = 1 + \displaystyle{{9\left( {\sum\nolimits_{i = 2}^n {{x_i}} } \right)} \over {n - 1}}} \cr } } \right.

where }{}0 \le {x_i} \le 1,\; \; i = 1,2, \ldots ,30. The Pareto optimal region corresponds to }{}{x_1} \in \left[ {0,1} \right] and }{}{x_i} = 0,\; i = 2,3, \ldots ,n.

The ZDT3 problem (Deb et al., 2002) is described as follows:

(19) }{}{\rm ZDT3}:\left\{ {\matrix{ {{\hskip-152pt}{\rm Minimize}\;{f_1}\left( x \right) = {x_1}} \cr {{\rm Minimize}\;{f_2}\left( x \right) = g\left( x \right)\left[ {1 - \sqrt {{x_1}/g\left( x \right)} - \displaystyle{{{x_1}} \over {g\left( x \right)}}\sin \left( {10{\rm \pi} {x_1}} \right)} \right]} \cr {g\left( x \right) = 1 + \displaystyle{{9\left( {\sum\nolimits_{i = 2}^n {{x_i}} } \right)} \over {n - 1}}} \cr } } \right.

where }{}0 \le {x_i} \le 1,\; \; i = 1,2, \ldots ,30. The Pareto optimal region corresponds to }{}{x_1} \in \left[ {0,1} \right] and }{}{x_i} = 0,\; i = 2,3, \ldots ,n.

The ZDT6 problem (Deb et al., 2002) is described as follows:

(20) }{}{\rm ZDT6:}\left\{ {\matrix{ {{\rm Minimize}\;{f_1}\left( x \right) = 1 - \exp \left( { - 4{x_1}} \right){\rm sin}{^6}\left( {6\pi {x_1}} \right)} \cr {{\hskip-18pt}{\rm Minimize}\;{f_2}\left( x \right) = g\left( x \right)\left[ {1 - {{\left( {\displaystyle{{{f_1}\left( x \right)} \over {g\left( x \right)}}} \right)}^2}} \right]} \cr {g\left( x \right) = 1 + 9{{{\left[ {\left( {\sum\limits_{i = 2}^n {{x_i}} } \right)/\left( {n - 1} \right)} \right]}^{0.25}}} \cr } }} \right.}

where }{}0 \le {x_i} \le 1,\; \; i = 1,2, \ldots ,10. The Pareto optimal region corresponds to }{}{x_1} \in \left[ {0,1} \right] and }{}{x_i} = 0,\; i = 2,\; 3,\; \ldots ,\; n.

The DTLZ1 problem (Deb et al., 2002) is described as follows:

(21) }{}{\rm DTLZ1:}\left\{ {\matrix{ {{\hskip-95pt}{\rm Minimize}\;{f_1}\left( x \right) = \displaystyle{1 \over 2}{x_1}{x_2} \ldots {x_{M - 1}}\left( {1 + g\left( {{x_M}} \right)} \right)} \cr {{\hskip-70pt}{\rm Minimize}\;{f_2}\left( x \right) = \displaystyle{1 \over 2}{x_1}{x_2} \ldots \left( {1 - {x_{M - 1}}} \right)\left( {1 + g\left( {{x_M}} \right)} \right)} \cr {\matrix{ \vdots  \vdots \cr } } \cr {{\hskip-95pt}{\rm Minimize}\;{f_{M - 1}}\left( x \right) = \displaystyle{1 \over 2}{x_1}\left( {1 - {x_2}} \right)\left( {1 + g\left( {{x_M}} \right)} \right)} \cr {{\hskip-110pt}{\rm Minimize}\;{f_M}\left( x \right) = \displaystyle{1 \over 2}\left( {1 - {x_1}} \right)\left( {1 + g\left( {{x_M}} \right)} \right)} \cr {{\hskip-127pt}{\rm subject\;to}\;0 \le {x_i} \le 1,i = 1,2, \ldots ,n} \cr {{\rm where}\;g\left( {{x_M}} \right) = 100\left( {\left| {{x_M}} \right| + \sum\limits_{{x_i} \in {x_M}} {{{\left( {{x_i} - 0.5} \right)}^2}} - \cos \left( {20\pi \left( {{x_i} - 0.5} \right)} \right)} \right)} \cr } } \right.

here, }{}M = 3, \left| {{x_M}} \right| = k = 5, the total number of variables is }{}n = M + k - 1. The Pareto-optimal solution corresponds to }{}x_i^* = 0.5\; \left( {x_i^* \in {x_M}} \right) and the objective function values lie on the linear hyper plane: \mathop \sum \nolimits_{m = 1}^M f_m^* = 0.5.

The DTLZ2 problem (Deb et al., 2002) is described as follows:

(22) }{}{\rm DTLZ2:}\left\{ {\matrix{ {{\rm Minimize}\;{f_1}\left( x \right) = \left( {1 + g\left( {{x_M}} \right)} \right)\cos \left( {\displaystyle{{{x_1}\pi } \over 2}} \right) \ldots \cos \left( {\displaystyle{{{x_{M - 1}}\pi } \over 2}} \right)} \cr {{\rm Minimize}\;{f_2}\left( x \right) = \left( {1 + g\left( {{x_M}} \right)} \right)\cos \left( {\displaystyle{{{x_1}\pi } \over 2}} \right) \ldots \sin \left( {\displaystyle{{{x_{M - 1}}\pi } \over 2}} \right)} \cr {\matrix{ \vdots  \vdots \cr } } \cr {{\hskip-60pt}{\rm Minimize}\;{f_M}\left( x \right) = \left( {1 + g\left( {{x_M}} \right)} \right)\sin \left( {\displaystyle{{{x_1}\pi } \over 2}} \right)} \cr {{\hskip-78pt}{\rm subject\;to}\;0 \le {x_i} \le 1,i = 1,2, \ldots ,n} \cr {{\hskip-97pt}{\rm where}\;g\left( {{x_M}} \right) = \sum\limits_{{x_i} \in {x_M}} {{{\left( {{x_i} - 0.5} \right)}^2}} } \cr } } \right.

here, }{}M = 3, \left| {{x_M}} \right| = k = 10, the total number of variables is }{}n = M + k - 1. The Pareto-optimal solution corresponds to }{}x_i^* = 0.5\; \left( {x_i^* \in {x_M}} \right) and the objective function values must satisfy the \mathop \sum \nolimits_{m = 1}^M {(f_m^*)^2} = 1.

The DTLZ3 problem (Deb et al., 2002) is described as follows:

(23) }{}{\rm DTLZ3:}\left\{ {\matrix{ {{\hskip-45pt}{\rm Minimize}\;{f_1}\left( x \right) = \left( {1 + g\left( {{x_M}} \right)} \right)\cos \left( {\displaystyle{{{x_1}\pi } \over 2}} \right) \ldots \cos \left( {\displaystyle{{{x_{M - 1}}\pi } \over 2}} \right)} \cr {{\hskip-45pt}{\rm Minimize}\;{f_2}\left( x \right) = \left( {1 + g\left( {{x_M}} \right)} \right)\cos \left( {\displaystyle{{{x_1}\pi } \over 2}} \right) \ldots \sin \left( {\displaystyle{{{x_{M - 1}}\pi } \over 2}} \right)} \cr {\matrix{ \vdots  \vdots \cr } } \cr {{\hskip-107pt}{\rm Minimize}\;{f_M}\left( x \right) = \left( {1 + g\left( {{x_M}} \right)} \right)\sin \left( {\displaystyle{{{x_1}\pi } \over 2}} \right)} \cr {{\hskip-125pt}{\rm subject\;to}\;0 \le {x_i} \le 1,i = 1,2, \ldots ,n} \cr {{\rm where}\;g\left( {{x_M}} \right) = 100\left( {\left| {{x_M}} \right| + \sum\limits_{{x_i} \in {x_M}} {{{\left( {{x_i} - 0.5} \right)}^2}} } \right) - \cos \left( {20\pi \left( {{x_i} - 0.5} \right)} \right)} \cr } } \right.

here, }{}M = 3, \left| {{x_M}} \right| = k = 10, the total number of variables is *n = M + k − 1*. The Pareto-optimal solution corresponds to }{}x_i^* = 0.5\; \left( {x_i^* \in {x_M}} \right).

The DTLZ6 problem (Deb et al., 2002) is described as follows:

(24) }{}{\rm DTLZ6:}\left\{ {\matrix{ {{\hskip-124pt}{\rm Minimize}\;{f_1}\left( x \right) = {x_1}} \cr {{\hskip-124pt}{\rm Minimize}\;{f_2}\left( x \right) = {x_2}} \cr \matrix{\matrix{ \vdots  \vdots \cr } \cr {\hskip-98pt}{\rm Minimize}\;{f_{M - 1}}\left( x \right) = {x_{M - 1}} } \cr {\matrix{ {{\rm Minimize}\;{f_M}\left( x \right) = \left( {1 + g\left( {{x_M}} \right)} \right)h\left( {{f_1},{f_2}, \ldots ,{f_{M - 1}},g} \right)} \cr {{\hskip-55pt}{\rm subject\;to}\;0 \le {x_i} \le 1,i = 1,2, \ldots ,n} \cr {\matrix{ {{\hskip-75pt}{\rm where}\;g\left( {{x_M}} \right) = 1 + \displaystyle{g \over {\left| {{x_M}} \right|}}\sum\limits_{{x_i} \in {x_M}} {{x_i}} } \cr {h = M - \sum\limits_{i = 1}^{M - 1} {\left[ {\displaystyle{{{f_i}} \over {1 + g}}\left( {1 + \sin \left( {3\pi {f_i}} \right)} \right)} \right]} } \cr } } \cr } } \cr } } \right.

here, *M* = 3, |x_*M*_| = *k* = 20, the total number of variables is *n = M + k − 1*.

### Experiment sets

To evaluate the performance of MOICSO for multi-objective problems, we compared it with MODA (Mirjalili, 2016) and MOWCA (Sadollah et al., 2015). In order to do meaningful statistical analysis, each algorithm runs for 10 times. The maximum evaluation count is 20,000. The population size of MOICSO was 100. The experimental results include the best value, the worst value, the mean value, the median value and the variance of the convergence measure and the diversity measure. Parameters for MOICSO are the same as ICSO in the section “Experiment and results of ICSO”. For the other specific parameters for involved algorithms, we can follow parameters settings of the original literatures of MODA (Mirjalili, 2016) and MOWCA (Sadollah et al., 2015).

### Experiment results and analysis

Figures 10–12 show the optimal front obtained by three algorithms on two objective test problems. The continuous solid line in the figure represents the Pareto optimal front of the theory, and the circle represents the non-dominated solution set obtained by each algorithm. Figures 13–20 show theoretical Pareto front and the optimal solution set obtained by three algorithms on three objective test problems.

**Figure 10:**
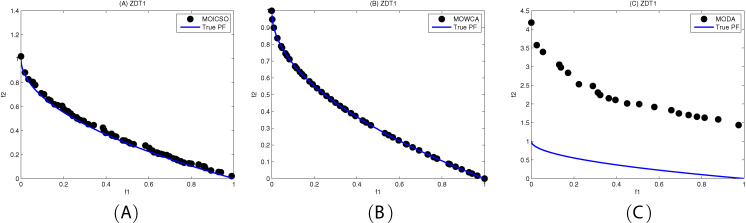
Pareto fronts obtained by (A) MOICSO, (B) MOWCA and (C) MODA on ZDT1 after 20,000 FEs.

**Figure 11:**
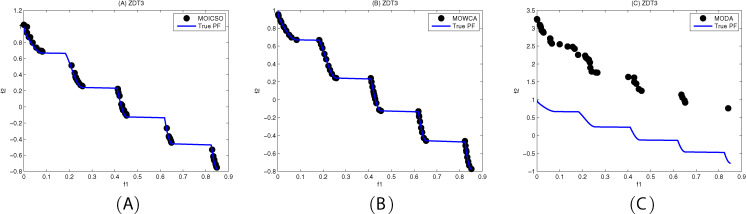
Pareto fronts obtained by (A) MOICSO, (B) MOWCA and (C) MODA on ZDT3 after 20,000 FEs.

**Figure 12:**
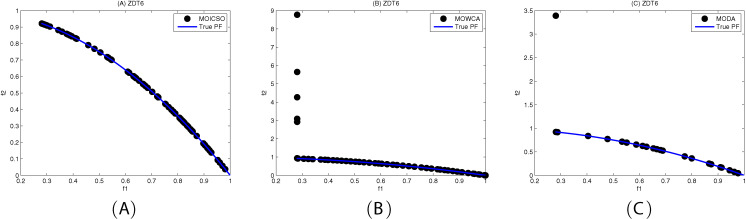
Pareto fronts obtained by (A) MOICSO, (B) MOWCA and (C) MODA on ZDT6 after 20,000 FEs.

**Figure 13:**
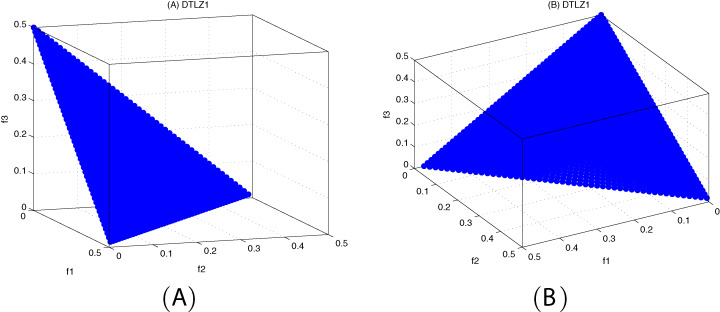
The true Pareto optimal front on DTLZ1. (A) Theoretical Pareto front on DTLZ1 with small elevation; (B) theoretical Pareto front on DTLZ1 with large elevation.

**Figure 14:**
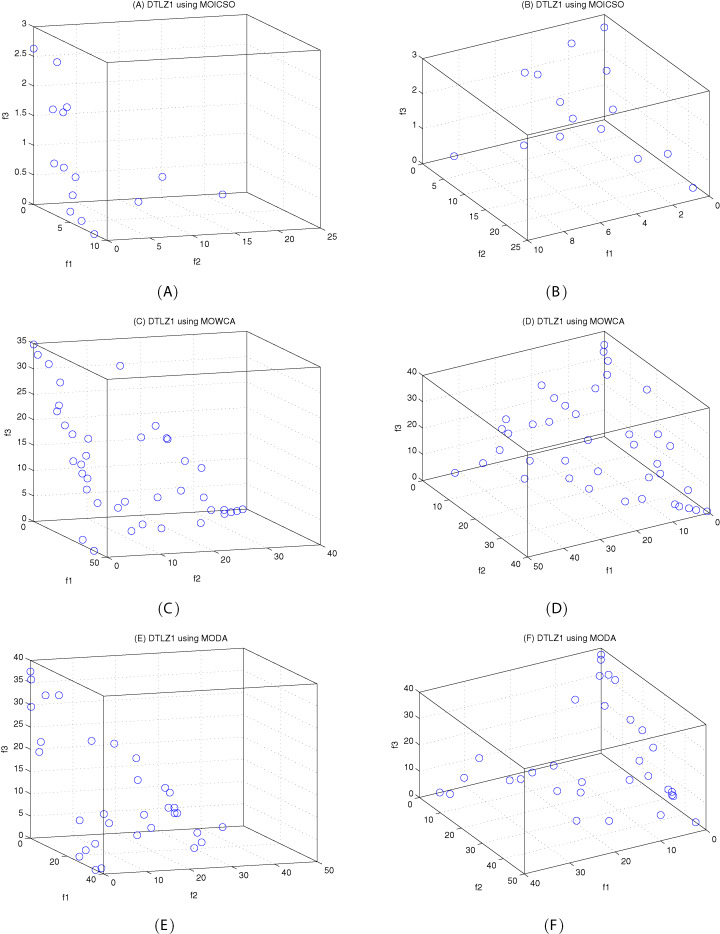
Pareto fronts obtained by MOICSO, MOWCA and MODA on DTLZ1 after 20,000 FEs. (A) Pareto front obtained by MOICSO on DTLZ1 with small elevation; (B) Pareto front obtained by MOICSO on DTLZ1 with large elevation; (C) Pareto front obtained by MOWCA on DTLZ1 with small elevation; (D) Pareto front obtained by MOWCA on DTLZ1 with large elevation; (E) Pareto fronts obtained by MODA on DTLZ1 with small elevation; (F) Pareto fronts obtained by MODA on DTLZ1 with large elevation.

**Figure 15:**
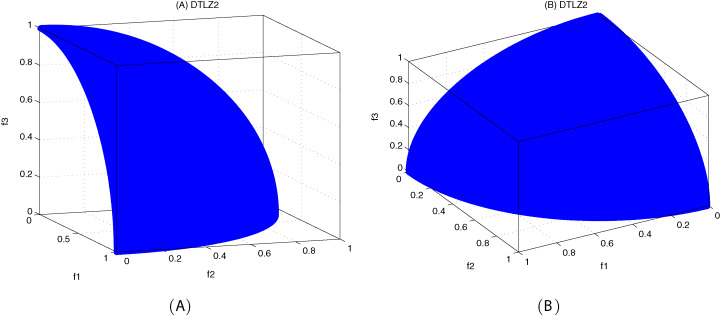
The true Pareto optimal front on DTLZ2. (A) Theoretical Pareto front on DTLZ2 with small elevation; (B) theoretical Pareto front on DTLZ2 with large elevation.

**Figure 16:**
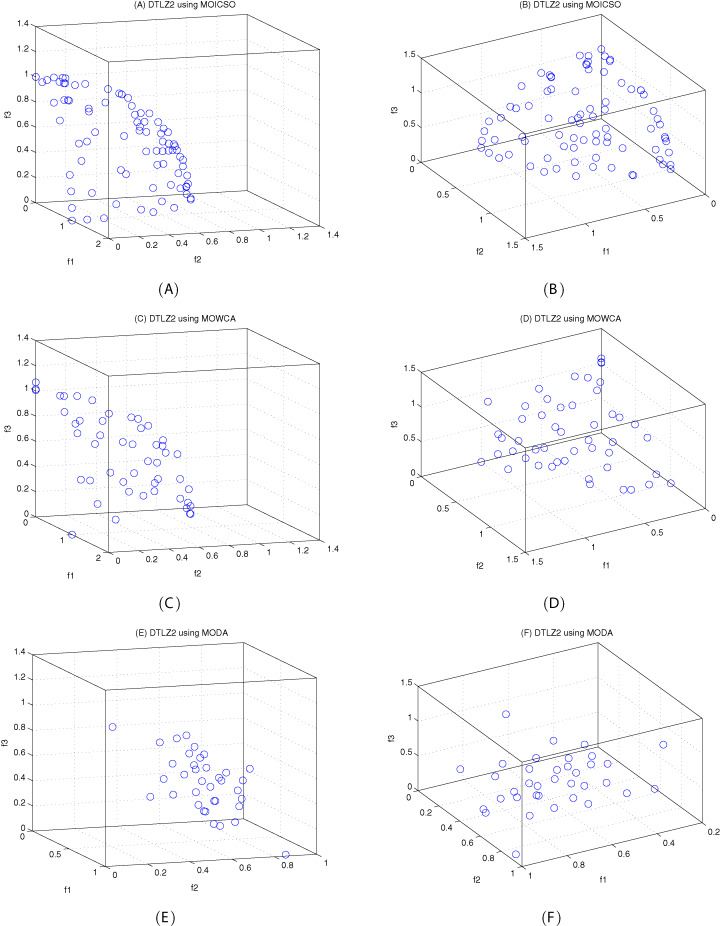
Pareto fronts obtained by MOICSO, MOWCA and MODA on DTLZ2 after 20,000 FEs. (A) Pareto front obtained by MOICSO on DTLZ2 with small elevation; (B) Pareto front obtained by MOICSO on DTLZ2 with large elevation; (C) Pareto front obtained by MOWCA on DTLZ2 with small elevation; (D) Pareto front obtained by MOWCA on DTLZ2 with large elevation; (E) Pareto fronts obtained by MODA on DTLZ2 with small elevation; (F) Pareto fronts obtained by MODA on DTLZ2 with large elevation.

**Figure 17:**
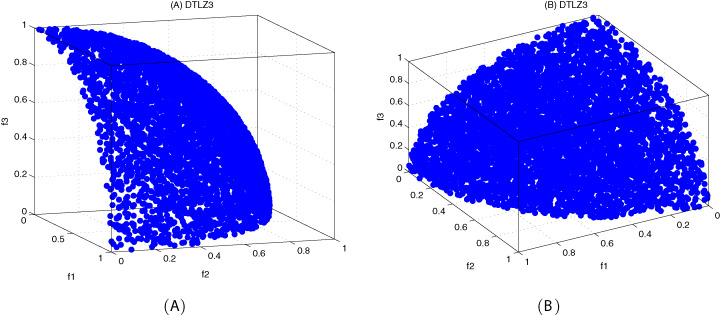
The true Pareto optimal front on DTLZ3. (A) Theoretical Pareto front on DTLZ3 with small elevation; (B) theoretical Pareto front on DTLZ3 with large elevation.

**Figure 18:**
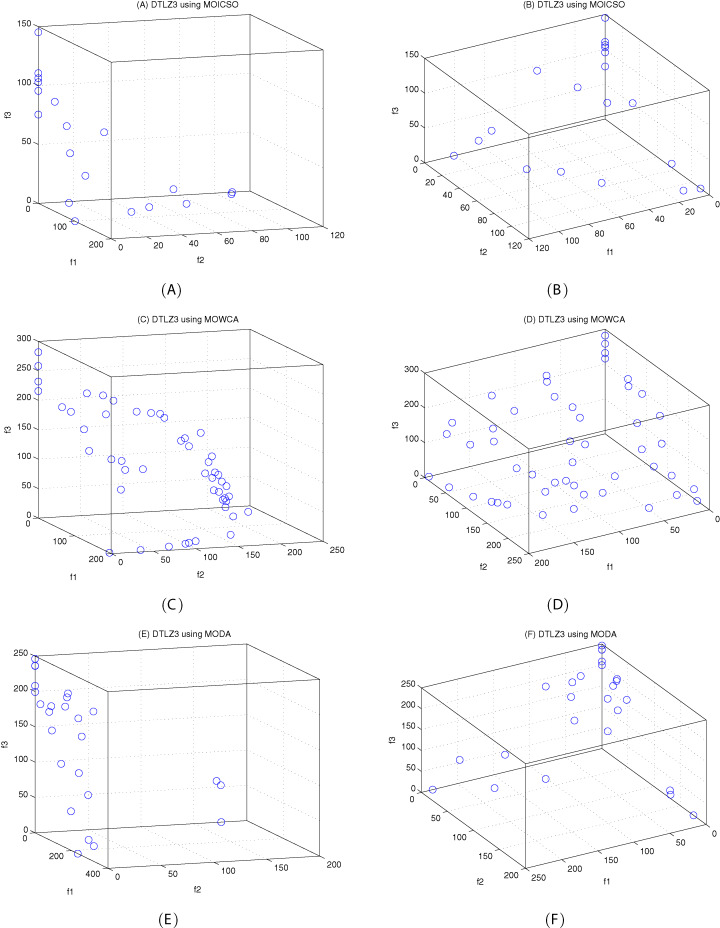
Pareto fronts obtained by MOICSO, MOWCA and MODA on DTLZ3 after 20,000 FEs. (A): Pareto front obtained by MOICSO on DTLZ3 with small elevation; (B) Pareto front obtained by MOICSO on DTLZ3 with large elevation; (C) Pareto front obtained by MOWCA on DTLZ3 with small elevation; (D) Pareto front obtained by MOWCA on DTLZ3 with large elevation; (E) Pareto fronts obtained by MODA on DTLZ3 with small elevation; (F) Pareto fronts obtained by MODA on DTLZ3 with large elevation.

**Figure 19:**
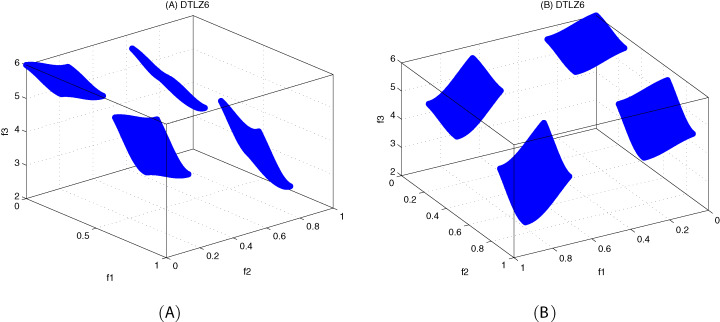
The true Pareto optimal front on DTLZ6. (A) Theoretical Pareto front on DTLZ6 with small elevation; (B) theoretical Pareto front on DTLZ6 with large elevation.

**Figure 20:**
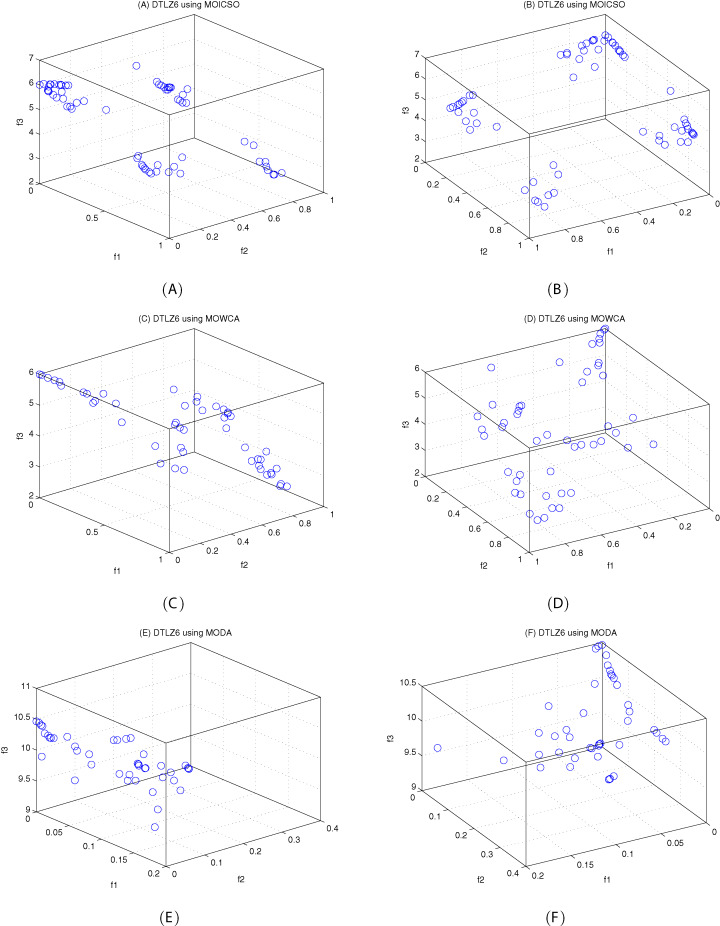
Pareto fronts obtained by MOICSO, MOWCA and MODA on DTLZ6 after 20,000 FEs. (A) Pareto front obtained by MOICSO on DTLZ6 with small elevation; (B) Pareto front obtained by MOICSO on DTLZ6 with large elevation; (C) Pareto front obtained by MOWCA on DTLZ6 with small elevation; (D) Pareto front obtained by MOWCA on DTLZ6 with large elevation; (E) Pareto fronts obtained by MODA on DTLZ6 with small elevation; (F) Pareto fronts obtained by MODA on DTLZ6 with large elevation.

For ZDT1 problem, it can be seen from Table 13 that MOICSO has better performance than MODA and MOWCA in diversity. However, MOWCA got the best performance in convergence. It can be seen from Fig. 10 that MODA cannot converge to the Pareto front of ZDT1.

**Table 13:** Comparison of performance on ZDT1.

ZDT1		MOICSO	MODA	MOWCA
GD	mean	2.217647E−02	1.613181E+00	**9.053588E−04**
	std	3.905024E−03	1.678708E−01	7.677344E−04
	median	2.190849E−02	1.634205E+00	6.617813E−04
	best	1.673679E−02	1.383347E+00	4.591891E−04
	worst	2.791896E−02	1.868831E+00	3.054644E−03
S	mean	**7.106517E−02**	8.570104E−02	7.877154E−02
	std	1.056309E−02	4.957961E−02	1.497220E−02
	median	7.246102E−02	6.432828E−02	8.170284E−02
	best	4.602081E−02	2.762794E−02	5.605837E−02
	worst	8.118060E−02	1.808153E−01	1.015106E−01

**Note:**

The best results are shown in bold.

For ZDT3 problem, it can be seen from Table 14 that MODA has better performance than MOICSO and MOWCA in diversity. However, it can be seen from Fig. 11 that MODA cannot converge to the Pareto front of ZDT3. MOWCA got the best performance in convergence. MOICSO outperforms MODA in terms of convergence and MOWCA in terms of diversity.

**Table 14:** Comparison of performance on ZDT3.

ZDT3		MOICSO	MODA	MOWCA
GD	mean	1.022275E−02	1.491569E+00	**3.577329E−03**
	std	3.252607E−03	2.222557E−01	4.373096E−04
	median	9.580086E−03	1.435797E+00	3.690424E−03
	best	7.326843E−03	1.184196E+00	2.813251E−03
	worst	1.784142E−02	1.817655E+00	4.139920E−03
S	mean	2.333496E−01	**1.132423E−01**	2.628808E−01
	std	2.914967E−02	6.452298E−02	2.986261E−02
	median	2.291432E−01	1.297423E−01	2.589863E−01
	best	1.933173E−01	2.833156E−02	2.205662E−01
	worst	2.833959E−01	2.301606E−01	3.201229E−01

**Note:**

The best results are shown in bold.

For ZDT6 problem, MOICSO outperforms the other two comparison algorithms in terms of convergence and diversity, as seen from Fig. 12 and Table 15. In particular, the performance of MOICSO in convergence metric is three order of magnitude better than that of MODA and MOWCA, as shown in Table 15. The performance of MOICSO in diversity metric is two order of magnitude better than that of MOWCA and one order of magnitude better than that of MODA, as shown in Table 15.

**Table 15:** Comparison of performance on ZDT6.

ZDT6		MOICSO	MODA	MOWCA
GD	mean	**2.611824E-03**	3.051035E+00	1.789835E+00
	std	3.330402E−04	2.073007E+00	7.875911E−01
	median	2.574583E−03	3.444935E+00	1.828042E+00
	best	2.000456E−03	5.574128E−03	6.014006E−02
	worst	3.086046E−03	5.195960E+00	2.701427E+00
S	mean	**7.100569E−02**	1.269278E−01	1.140027E+00
	std	7.083846E−03	1.230177E−01	6.900851E−01
	median	7.213973E−02	8.527509E−02	1.010902E+00
	best	5.959513E−02	1.231576E−02	1.105152E−01
	worst	8.493150E−02	3.902315E−01	2.821910E+00

**Note:**

The best results are shown in bold.

For DTLZ1 problem, it can be seen from Fig. 14 and Table 16 that MOICSO has better performance than MODA and MOWCA in both convergence and diversity.

**Table 16:** Comparison of performance on DTLZ1.

DTLZ1		MOICSO	MODA	MOWCA
GD	mean	**5.599968E+00**	2.723070E+01	3.087193E+01
	std	2.094972E+00	1.724426E+00	1.768829E+01
	median	4.939401E+00	2.760911E+01	3.001736E+01
	best	2.618534E+00	2.404774E+01	1.059995E+01
	worst	9.806825E+00	2.896706E+01	7.264215E+01
S	mean	**3.683550E+00**	6.019641E+00	6.690146E+00
	std	2.043435E+00	1.532413E+00	8.730985E+00
	median	3.444867E+00	5.428381E+00	4.473351E+00
	best	1.120093E+00	4.209876E+00	5.363014E−01
	worst	7.595202E+00	8.757288E+00	3.016426E+01

**Note:**

The best results are shown in bold.

For DTLZ2 problem, it can be seen from Fig. 16 and Table 17 that MOICSO outperforms the other two comparison algorithms in convergence. However, MODA obtains the best result among all the algorithms in diversity.

**Table 17:** Comparison of performance on DTLZ2.

DTLZ2		MOICSO	MODA	MOWCA
GD	mean	**4.235746E−03**	1.542991E−01	5.190870E−02
	std	2.142640E−04	4.563576E−02	8.397780E−03
	median	4.135215E−03	1.487712E−01	4.940963E−02
	best	3.999748E−03	7.502808E−02	4.269642E−02
	worst	4.616637E−03	2.359028E−01	6.732258E−02
S	mean	1.731054E−01	**6.471356E−02**	1.908362E−01
	std	1.436543E−02	2.918751E−02	1.938634E−02
	median	1.770953E−01	6.830215E−02	1.969874E−01
	best	1.491236E−01	2.626786E−02	1.569525E−01
	worst	1.937718E−01	1.090731E−01	2.190289E−01

**Note:**

The best results are shown in bold.

For DTLZ3 problem, it can be seen from Fig. 18 and Table 18 that MOICSO outperforms the other two comparison algorithms in both convergence and diversity. In particular, the performance of MOICSO in convergence metric is one order of magnitude better than that of MODA and MOWCA.

**Table 18:** Comparison of performance on DTLZ3.

DTLZ3		MOICSO	MODA	MOWCA
GD	mean	**9.460404E+01**	2.108529E+02	2.242903E+02
	std	1.122988E+01	2.109525E+01	3.206016E+01
	median	9.392081E+01	2.078806E+02	2.204787E+02
	best	7.682157E+01	1.777625E+02	1.741408E+02
	worst	1.137845E+02	2.424980E+02	2.773862E+02
S	mean	**2.507854E+01**	5.137025E+01	5.264536E+01
	std	1.214376E+01	1.548518E+01	4.150575E+01
	median	2.167307E+01	5.419331E+01	4.200310E+01
	best	9.280821E+00	2.081172E+01	2.234076E+01
	worst	5.498289E+01	7.131771E+01	1.680020E+02

**Note:**

The best results are shown in bold.

For DTLZ6 problem, it can be seen from Fig. 20 and Table 19 that MOICSO outperforms the other two comparison algorithms in terms of convergence. MODA obtains the best result among all the algorithms in diversity.

**Table 19:** Comparison of performance on DTLZ6.

DTLZ6		MOICSO	MODA	MOWCA
GD	mean	**1.741554E−02**	5.178208E+00	6.917079E−02
	std	4.430565E−03	8.785128E−01	1.115014E−02
	median	1.686732E−02	5.204424E+00	6.513040E−02
	best	1.191828E−02	3.801288E+00	5.510898E−02
	worst	2.597976E−02	6.766510E+00	8.426057E−02
S	mean	4.001566E−01	**2.757844E−01**	5.470521E−01
	std	6.900276E−02	1.642457E−01	7.785121E−02
	median	4.136595E−01	3.180918E−01	5.373484E−01
	best	2.962141E−01	4.524112E−02	4.236813E−01
	worst	5.258488E−01	4.468865E−01	6.576980E−01

**Note:**

The best results are shown in bold.

## Conclusion

Most of real problems such as tuning proportional integral derivative (PID) controller parameters and scheduling problem are uncertain, dynamic, nonlinear, multi-modal or NP-hard problems. PID controller has been widely used in industry for many years. However, the performance of the systems with standard PID controllers cannot meet the design requirements because of the nonlinear dynamics, strong external disturbance and the random noise (Tang, Zhang & Hu, 2020). When tuning PID controller parameters, it is difficult to obtain the optimal or near optimal PID parameters by using some traditional tuning methods. The permutation flow-shop scheduling problem (PFSP) has been extensively explored in production scheduling. As the PFSP is NP-hard even for the single-objective problem (Gonzalez & Sahni, 1978). Recently, meta-heuristic algorithm provides novel approaches for solving complicated problems as mentioned above. This article proposed an ICSO which is a hybrid meta-heuristic algorithm. In order to avoid the shortcomings of single algorithms, ICSO integrates heterogeneous biological-inspired strategies and mechanisms into one algorithm. These strategies include a unique two-subpopulation structure, an adaptive variable step size mechanism based a linear decreasing function and a crossover based on DE operation. With the iteration of the algorithm, the size of the two subpopulations changes dynamically, but the size of the whole population remains unchanged. Furthermore, the individual fitness information in the optimization process is used to divide the two subpopulations. The two subpopulations adopt different optimization methods. The high fitness subpopulation is responsible for exploiting better solutions, while the low fitness subpopulation is responsible for exploring unknown solutions. The diversity of solutions is increased due to the different optimization methods adopted by the two subpopulations. In order to exchange information between individuals, the two subpopulations are merged after iteration and a crossover operation based on DE is applied.

In order to see how ICSO performs, a series of experiments on 24 classic standard functions compared with DDICS, CSDE, CSEI and DE/rand are carried out. The comprehensive analysis of ICSO shows that ICSO yielded better results than the other comparison methods with the same number of evaluations and runs on most cases. The paper also considers the proposal of multi-objective versions of ICSO called MOICSO. Based on the test problems considered and compared with other latest methods, the optimization results show the robustness and effectiveness of MOICSO for solving multi-objective optimization problems. ICSO is an effective method for unstrained optimization problems, so ICSO being applied to a variety of complex real-world problems is the future work.

## Supplemental Information

10.7717/peerj-cs.370/supp-1 Supplemental Information 1 Code for ICSO.

## Additional Information and Declarations

### Data Availability

The following information was supplied regarding data availability: The code is available in the Supplemental Files.
